# Post-stroke unilateral spatial neglect: virtual reality-based navigation and detection tasks reveal lateralized and non-lateralized deficits in tasks of varying perceptual and cognitive demands

**DOI:** 10.1186/s12984-018-0374-y

**Published:** 2018-04-23

**Authors:** Tatiana Ogourtsova, Philippe S. Archambault, Anouk Lamontagne

**Affiliations:** 1 School of Physical and Occupational Therapy, McGill University, 3654 Promenade Sir-William-Osler, Montreal, Quebec H3G 1Y5 Canada; 2 Feil-Oberfeld Research Centre, Jewish Rehabilitation Hospital, Laval, Quebec Canada

## Abstract

**Background:**

Unilateral spatial neglect (USN), a highly prevalent and disabling post-stroke impairment, has been shown to affect the recovery of locomotor and navigation skills needed for community mobility. We recently found that USN alters goal-directed locomotion in conditions of different cognitive/perceptual demands. However, sensorimotor post-stroke dysfunction (e.g. decreased walking speed) could have influenced the results.

Analogous to a previously used goal-directed locomotor paradigm, a seated, joystick-driven navigation experiment, minimizing locomotor demands, was employed in individuals with and without post-stroke USN (USN+ and USN-, respectively) and healthy controls (HC).

**Methods:**

Participants (*n* = 15 per group) performed a seated, joystick-driven navigation and detection time task to targets 7 m away at 0°, ±15°/30° in actual (visually-guided), remembered (memory-guided) and shifting (visually-guided with representational updating component) conditions while immersed in a 3D virtual reality environment.

**Results:**

Greater end-point mediolateral errors to left-sided targets (remembered and shifting conditions) and overall lengthier onsets in reorientation strategy (shifting condition) were found for USN+ vs. USN- and vs. HC (*p* < 0.05). USN+ individuals mostly overshot left targets (− 15°/− 30°). Greater delays in detection time for target locations across the visual spectrum (left, middle and right) were found in USN+ vs. USN- and HC groups (*p* < 0.05).

**Conclusion:**

USN-related attentional-perceptual deficits alter navigation abilities in memory-guided and shifting conditions, independently of post-stroke locomotor deficits. Lateralized and non-lateralized deficits in object detection are found. The employed paradigm could be considered in the design and development of sensitive and functional assessment methods for neglect; thereby addressing the drawbacks of currently used traditional paper-and-pencil tools.

**Electronic supplementary material:**

The online version of this article (10.1186/s12984-018-0374-y) contains supplementary material, which is available to authorized users.

## Background

Unilateral spatial neglect (USN) is a highly disabling disorder, that is present in at least 30% of all stroke survivors [1] and in nearly 50% of individuals with right hemisphere lesions following a stroke [2]. USN is characterized by a decrease in orientation and/or response time to contralesionally located stimuli [3]. It is known to persist into the chronic stages of stroke recovery, poorly respond to available treatment methods, and significantly contribute to functional deterioration (reviewed in [4]) and reduced quality of life [5] of the affected individuals.

One of the most sought rehabilitation goals among stroke survivors is to regain independent mobility within the community environments [6], as safe and efficient locomotion and/or navigation in space is necessary for numerous self-care and instrumental activities of daily life. Alas, less than 40% of individuals with post-stroke USN regain independent walking abilities [7]. Consequently, it is paramount to investigate the role of spatial cognition on locomotion and navigation in individuals with post-stroke USN, with a general aim to improve rehabilitation practice in that field and ameliorate patient-related health outcomes. Yet, the literature addressing the effects of USN on walking and/or navigation abilities remains limited and necessitates further investigation before practice recommendations can be implemented (e.g. [8–13]). Up to now, studies reported deviations of walking/navigation trajectories in patients with post-stroke USN [8–11, 14, 15], as well as collisions with stationary [11, 14, 15] and moving obstacles [12, 16]. Our team has recently demonstrated that individuals with post-stroke USN vs. stroke individuals without USN show defective goal-directed walking abilities when heading towards left located (contralesional/ “neglected”) and right located (ipsilesional/“non-neglected”) targets in conditions of variable cognitive/perceptual demands: where the visual target could remain stationary, disappear or shift position during walking. Nevertheless, participants with USN walked considerably slower compared to those without USN, making walking speed a potential confounding factor when comparing the performance of the two groups. Therefore, whether goal-directed walking deficits result from perceptual-attentional deficits caused by USN or are also mediated by post-stroke sensorimotor dysfunctions which affects gait, balance and posture, remains unresolved and warrants investigation. To further our understanding of the role of post-stroke USN in the control of goal-directed walking, we propose to examine its effects in a joystick-driven goal-directed navigation task which is analogous to the goal-directed walking tested earlier [13]. The main premise behind this paradigm is that the use of a joystick for navigation with the non-paretic hand, performed in sitting, minimizes the biomechanical demands of locomotion and its concurrent sensorimotor aspects. Thus, it permits to essentially examine the role of attentional-perceptual abilities involved by eliminating potential confounding factors related to gait capacity, such as walking speed. In addition, the proposed joystick-driven seated task represents a more feasible approach (to assess certain aspects of mobility in post-stroke individuals in comparison to a goal-directed locomotion task, that requires more resources (in terms of equipment, space/setup and timing). Therefore, the joystick-driven task could potentially be more suitable to be implemented in the clinical setting.

The navigation scene and conditions employed in this study were analogous to a previously conducted goal-directed locomotor experiment [13] and included three conditions: navigation to an actual target (always present and visible to the participant, online condition); navigation to a remembered target (present at first then disappears during navigation, offline condition); and navigation to a shifting target (changes location following forward displacement of the participant, online condition). The primary objective of this study was to estimate the extent to which post-stroke USN affects goal-directed navigation abilities in online and offline conditions. Secondary objectives were to estimate the extent to which post-stroke USN affects target detection abilities, what is the relationship of navigation abilities with measures of detection abilities and clinical measures of USN, and whether the navigation task can detect USN-related deficits that were otherwise left undetected using conventional methods. We hypothesized that post-stroke USN would affect navigation and detection abilities, such that greater end-point accuracy errors, longer re-orientation of navigation trajectories and greater detection times would be observed for the group with USN vs. those without USN and healthy controls, possibly in all conditions. We also hypothesized that clinical USN measures and target detection abilities would be minimally associated with navigation outcomes. In addition, we speculated that the navigation task in more cognitively/perceptually demanding conditions (i.e. remembered and shifting vs. actual) would be sensitive in detecting deficits that were otherwise left undetected using conventional paper and pencil USN assessment tools.

## Methods

### Participants

Fifteen individuals (*n* = 15) per group were recruited, tested and analyzed for the study. Individuals with stroke were included based on the following criteria: (1) presence of a first-time right hemisphere stroke (as per computer tomography (CT) report, neurological examination, and medical chart), (2) with or without left USN (as per one or more of the following tests: Line Bisection Test (LBT) [17], Star Cancellation Test (SCT) [18], and/or Apples Test (APT) [19] on testing, or history of USN as per medical chart); (3) age between 40 and 85 years old; (4) right handedness (as per interview and/or medical chart containing Edinburgh Handedness Inventory scores [20]). Given that participants were also involved in a walking experiment, they were all walking independently with or without a walking aid over a minimal distance of 5 m. Individuals were excluded based on the following criteria: (1) presence of primary visual impairment that impedes normal or corrected-to-normal binocular visual acuity (score ≤ 20/20 on the Early Treatment Diabetic Retinopathy Study Chart [21]); (2) presence of moderate cognitive impairment (score ≤ 22/30 the Montreal Cognitive Assessment [22]); (3) presence of a documented visual field deficits (Goldmann perimetry or computerized equivalent, as per medical chart); and (4) any premorbid neurological and/or orthopedic condition that can impede locomotion. Age–matched (±5 years) healthy controls were also recruited, following the same inclusion/exclusion criteria (where applicable).

Participants with (USN+) and without (USN-) post-stroke USN were recruited from the inpatient discharge lists of three clinical sites of Centre de Recherche Interdisciplinaire en Réadaptation du Montreal Métropolitain (CRIR), including the Jewish Rehabilitation Hospital (JRH), Centre de Réadaptation Lucie Bruneau and the Institut de Réadaptation Gingras-Lindsay de Montréal. These sites provide inpatient and outpatient post-stroke rehabilitation for patients living in the Greater Montreal area, Quebec, Canada. Healthy controls (HC) were recruited from the research database of the JRH and word-of-mouth using snowball sampling technique. Pre-authorized advertisement in form of wall-mounted notice was also used to recruit participants. All study participants provided their informed consent before enrolling in the study, as approved by the CRIR Institutional Review Board (CRIR-935-0214).

### Data collection

The process of data collection consisted of (1) clinical USN evaluation followed by the (2) virtual reality (VR)-based goal-directed navigation and detection time tasks. Experiments were carried out in a single session of approximately 30–45 min (including set-up time). In addition, prior to experimental data collection, each participant also completed a set of clinical measures of walking speed (10-Meter Walk Test (10MWT) [23–25]), mobility (Rivermead Mobility Index (RMI) [26]), and post-stroke recovery of lower extremities motor function (Chedoke McMaster Stroke Assessment (CMSA) - Leg and Foot [27]).

### Clinical USN evaluation

#### Apparatus and stimuli

Presence, severity and type of USN were determined using the LBT, SCT, and the APT which all show excellent psychometric properties [19, 28, 29]. The LBT and SCT were repeated in the near and far extrapersonal space, using a procedure previously employed, where participants were positioned 40 cm and 320 cm away from the screen for near and far USN testing, respectively. [8, 30]. Both tests were projected on the screen with appropriate sizes (near space: 21 × 28 cm; far space: 168 × 224 cm) to keep the visual angle of each array and the retinal size image constant during the two testing conditions. Responses were provided with a laser. The APT was presented on a sheet of paper on a steady table, aligned with the participant’s midline (i.e. sternum) and fixed on the table with tape to prevent possible shifts.

#### Procedure

In the LBT, participants were asked to find the midline of each presented line (*n* = 18), starting from the top line. In the SCT, participants were instructed to find all the small stars (*n* = 52) among the distractors. In the APT, participants were instructed to find all the whole apples (*n* = 50). An absolute mean deviation of more than 6.0 mm [17], and 4.8 cm to the right is indicative of left near-space and far-space USN, respectively. An average percentage of deviation from midline was also computed for near and far-space LBT to estimate the difference in severity between near and far space USN. Scores between 0 and 0.46 are indicative of left near-space USN on the SCT, computed as the number of crossed out small start over the total number of small stars [17]. In the APT, the total number of crossed out complete and incomplete apples was computed, and an asymmetry scores for egocentric (i.e. difference between the numbers of complete shape targets crossed out on the right versus left side of the page) and allocentric (i.e. difference between the numbers of incomplete shape targets crossed out with a right and with a left opening) USN were calculated [19]. The overall cutoff of < 42/50 is indicative of near-space USN. Asymmetry cutoff score across the page of <− 2 or > 2 (difference between right side and left-sided targets cancelled) is indicative of egocentric near-space USN. Asymmetry cutoff score across the cancelled distractors on the page with left vs right sided openings of <− 1 or > 1 is indicative of allocentric near-space USN. All cancellation tests were timed.

Severity of USN was characterized by a positive result on 1 to 3 (mild), 4 (moderate), and 5 or more (severe) clinical test scores out of 7. This classification was modified from Lindell et al. (2007) for mild (positive result on 1–3) vs. moderate/severe USN (positive result on 4 or more tests) [31] to distinguish moderate vs. severe cases.

#### Outcomes

Outcomes retained for analysis included: overall USN severity (history, mild, moderate or severe), (2) USN range of space severity (near and/or far-space), and (3) USN spatial representation type (allocentric vs egocentric). Participants were included in the USN+ group if they had USN on one or several of the aforementioned tests, or if they had a history of USN as per their medical chart. Subjects with a history of neglect are individuals who had neglect on clinical USN measures in the acute and/or subacute phases of stroke recovery but who no longer show neglect on clinical measures when in the chronic phase, that is at the time when they were recruited in the study. The reasoning behind this inclusion criteria is that previous studies showed deficits consistent with USN presentation among individuals with history of USN [15, 16, 32–35], possibly due to a poor sensitivity of clinical USN tests and/or because deficits may become more apparent when executing more functional and complex activities.

### VR-based goal-directed navigation and detection time tasks

#### Apparatus and stimuli

**Fig. 1:**
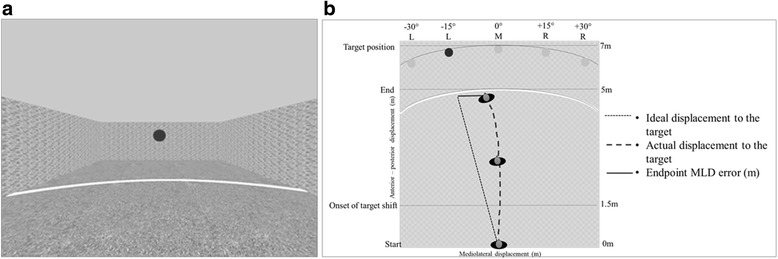
VR scene used in the present experiment. **a**. The VR scene used in the experiment. The target (i.e. red ball) appeared 7 m away from the starting position and at the following 5 possible locations: ±15°/30°, 0° (centered target location illustrated). Navigation trials were performed while immersed in the VR scene under actual, remembered and shifting target conditions **b**. Bird-eye view of the VR scene illustrating the start position (0 m), the 5 possible target locations (7 m radius from start position), onset distance for target shift in the shifting target condition (1.5 m) and endpoint position (5 m radius from start position). Outcomes measures endpoint mediolateral displacement (MLD) error is shown for a navigation trial to the left target at − 15°

The VR-based navigation and detection tasks were performed while seated and immersed in a 3-D virtual environment (VE) representing a symmetrical and richly-textured room (9 m × 15 m) including a visual display of walls and ceiling (i.e. giving an impression of indoor space with appropriate depth cues [36]). The target, a red ball, was presented 7 m away from the starting position (i.e. far-space) and at the following 5 possible locations: ±15°/30°, 0° (Fig. 1a). The target appeared at the same height and size in the visual field to avoid differences in distance perception [37, 38]. The VE scene was created in Softimage XSI®. During the experiments, the real-time CAREN-3™ (Computer Assisted Rehabilitation Environment; Motek BV, Amsterdam) software was employed to control the scene. The viewing media was a helmet mounted display (HMD - NVisor™, NVIS Inc., Reston, VA, USA) with a binocular field of view of 60° diagonal, 30° vertical by 40° horizontal, Extended Graphics Array resolution (1024 × 1280 pixels), refresh frequency of 60 Hz, 1 kg in weight, and blocking all peripheral vision with only the VE visible to the participant. Responses were provided with the dominant, non-paretic right hand using a joystick (Attack3™, Logitech, Newark, CA, USA), securely fixed on a table at a comfortable height, adjusted for each participant. The joystick controlled a pointer (not visible to the participant) that represented the position of the individual in first-person view. The VE scene was viewer-centered and the HMD real time tracking was disabled, allowing the scene to remain stable and viewer-centered, regardless of head orientation. Navigation in the scene, when required, was possible using the joystick in the mediolateral (left/right) planes at constant and pre-set speed of 0.75 m/s, being the average speed of ambulatory stroke population [10].

#### Procedure

##### Goal-directed navigation

Practice trials were performed (*n* = 5–10) prior the actual experiment until the participant felt comfortable in executing the task. For each condition (actual, remembered and shifting), five trials per target location (±30̊ ±15̊, 0°) were performed, for a total of 75 navigation trials or 25 trials per condition. Condition and target location were randomized. Prior to each trial, a “GET READY” sign appeared. At the end of each navigation trial, a “STOP” sign appeared; following which, the scene was recalibrated to the starting position for the beginning of a new trial. While the target was at 7 m away from the starting position, the navigation trial ended at 5 m of forward displacement, to avoid the cursor hitting the target (Fig. 1b).

In all conditions, a single target first appeared on the screen for 2000 ms. This was followed by a beep sound, signalling participants to navigate towards the target using the joystick. In the *actual* condition, the target remained visible during movement while in the *remembered* condition, it disappeared after the beep. Finally, in the *shifting* condition, the target remained visible after the beep but following 1.5 m of forward displacement, it could either shift its location to the right or left (+ 15°/30̊ or − 15°/30̊) or remain in the middle. If the shift occurred, participants were instructed to re-orient their navigation towards the new target location as soon as possible.

##### Detection task

The target appeared at the following 5 possible angles (±30̊, ±15, 0°) with randomized onset times. Participants were instructed to press the front joystick button with their index finger of the non-paretic, dominant, right hand as soon as they perceived the target. Catch trials (*n* = 10 within each condition) lasting 2500 ms, with no target, were introduced to minimize response bias. Five trials per location were performed for total of 75 trials. Target location was randomized between trials.

##### Outcomes

Outcomes related to the goal-directed navigation and detection tasks included the following: (1) *Endpoint mediolateral displacement (MLD) error* (i.e. accuracy measure, m), defined as the difference in meters between the mediolateral position of the target and that of the individual at 5 m of forward displacement; (2) *Direction of trajectory deviation,* defined as the side (left or right) from the target where the individual was located at the end of the trial; (3) *Onset of reorientation strategy* (for shifting condition only, i.e. temporal measure, sec), was determined using a variation of the extrapolation method [39], based on the extrapolation of trajectory segments. First, movement trajectory segments before (i.e. control movement) and after (i.e. adjusted movement) the shift were outlined and fitted using linear regression. Following, a line between 15 and 85% of the fitted trajectory was outlined and extrapolated. The onset of reorientation strategy is thus defined by the time at which the target was shifted (i.e. at 1.5 m of forward displacement) minus the time at which the extrapolated lines for pre- and post-shift crossed each other (Additional file 1); and (4) *Detection time* (temporal measure, sec), defined as the time difference between target appearance and its detection by the participant.

### Data and statistical analyses

All data were recorded at 120 Hz in CAREN-3™ and stored for off-line analyses in Matlab 2016a (The Mathworks, USA). Participants’ responses on goal-directed navigation and target detection tasks were averaged across conditions (navigation task) and target locations (navigation and detection tasks), such that mean values could later be compared across groups and between conditions. All statistical analyses were performed using SAS 9.4 (SAS Institute Inc). Significance was accepted at *p* ≤ 0.05.

The effects of post-stroke USN, target condition and target location on goal-directed navigation performances were examined using repeated measures mixed-model analysis, with ‘Group’ [USN+, USN-, HC] as between subject factor x ‘Target Condition’ [actual vs. remembered vs. shifting] x ‘Target Location’ [±15°/30°, 0°] as within subject factors. In the event of significant 3-way interaction effect of Group x Target Condition x Target Location, post-hoc comparisons of simple effects were elaborated on using previously identified relevant pairwise comparisons and included 1) within USN+/USN-/HC groups comparisons with target condition and target location factors (e.g. responses to − 30° target in actual vs. -30° target in remembered condition within the USN+ group); and 2) between USN+/USN-/HC group comparisons for each angle and condition (e.g. responses to − 30° target in actual condition for USN+ vs. USN- groups).

Further, to estimate the effect of post-stroke USN on the delay in re-orientation strategy to the left [− 15°/− 30° target shift] and to the right [+ 15°/+ 30° target shift] sides were examined separately for each side using the repeated-measures mixed-model analysis, with ‘Group’ [USN+ vs. USN- vs. HC] as the between-subject factor.

Following, the effects of USN and target location on detection times were examined using repeated-measures mixed-model analysis, with ‘Group’ [USN+ vs. USN- vs. HC] as a between subject factor x ‘Target Location’ [±15°/30°, 0°] as a within subject factor.

Kendall rank correlation coefficients were used to quantify the relationship of goal-directed navigation performances to the left (− 30°) target with clinical assessments of neglect within near and far spaces (LBT, SCT, APT) and detection time outcome to the left-sided target (− 30°) given that the data was not normally distributed in the USN+ group. The size of the correlation coefficient was interpreted as per established guidelines: very high (0.90–1.00), high (0.70–0.90), moderate (0.50–0.70), low (0.30–0.50) or negligible (0.00–0.30) [40].

To determine whether the navigation task can detect deficits otherwise not identified using traditional tests, single case analyses were used to compare the performance of each participant with history of USN as well as to compare the performance of each USN- participant with respect to the average performance of the HC group. Precisely, the Crawford and Garthwaite (2002) approach (Singlims.exe, University of Aberdee, Aberdeen, UK) [41], implementing classical methods for comparison of a single case’s score to scores obtained in a control sample, was used. This approach tests whether an individual’s score is significantly different from a control or normative sample. Results provide a point estimate of the effect size for the difference between the case and controls with an accompanying 95% confidence interval, and a point and interval estimate of the abnormality of the case’s score, where it estimates the percentage of the population that would obtain a lower score.

## Results

Fifteen individuals with post-stroke USN (USN+, *n* = 15, 60.2 ± 8.8 years old, 1.6 ± 1 year post-stroke, 11/15 ischemic stroke, 12 males), fifteen individuals post-stroke without USN (USN-, *n* = 15, 58.5 ± 13.2 years old, 2.0 ± 2.1 years post-stroke, 13/15 ischemic stroke, 13 males), and fifteen age-matched healthy control individuals (HC, *n* = 15, 61.0 ± 11.8 years old, 7 males) were recruited in the period between December 2014 and March 2016 (Table 1). Each participant successfully completed all the experimental trials. Both the USN+ and USN- groups predominantly consisted of male participants and were statistically similar in terms of stroke chronicity. No significant between-group differences were found on all baseline characteristics across the 3 study groups; with exception of walking speed where USN+ individuals were slower walkers vs. USN- and HC groups (*p* < 0.05) Table 1.

**Table 1:** Descriptive variables of study groups

Participant	Sex	Age	Stroke Chronicity	Type of Stroke	Stroke Location	10 MWT Fast/Comfortable	CMSA	RMI
	(M/F)	(years)	(years)			(m/s)	Leg/Foot	
USN+ (*n* = 15)								
1	M	53.5	4.1	Ischemic	P-T	1.5/1.0	6/6	14
2	M	72.3	2.7	Ischemic	Sylvian	1.2/0.9	6/6	13
3	M	45.7	1.6	Hemorrhagic	P	2.1/1.5	7/7	15
4	M	57.2	3.5	Hemorrhagic	P-T	1.7/1.4	7/7	14
5	M	69.2	1.3	Ischemic	P-O	1.7/1.1	7/6	14
6	M	51.5	1.6	Hemorrhagic	P-T	1.8/1.3	7/7	15
7	M	54.3	0.8	Ischemic	Periventricular and cerebral peduncle	1.3/0.5	6/6	14
8	F	69.0	0.9	Hemorrhagic	Subarachnoid hemorrhage grade 3, common right artery	1.6/1.2	7/7	15
9	M	67.7	1.3	Ischemic	Pontocerebellar fibers	2.1/1.4	7/7	15
10	F	50.8	2.5	Ischemic	P-T	0.7/0.6	5/5	12
11	M	61.6	0.9	Ischemic	P-O	1.5/1.2	6/6	15
12	M	73.1	0.3	Ischemic	P-O + midline shift	1.5/1.2	6/6	14
13	M	67.6	1.5	Ischemic	F-P	0.9/0.7	6/6	13
14	M	53.7	0.9	Ischemic	F-T-Ins	0.3/0.3	5/1	10
15	F	56.5	1.3	Ischemic	Sylvian	0.3/0.3	6/5^a^	12
Mean ± SD Range (−) Ratio (:)	12:3	60.2 ± 8.8	1.6 ± 1.0	11:4	NA	1.3 ± 0.5 *§/0.9 ± 0.4 *§	5–7 / 1–7	10–15
USN- (*n* = 15)								
16	M	50.6	0.5	Ischemic	P-O	1.6/1.3	7/7	15
17	F	81.1	1.7	Ischemic	F-P	1.3/1.0	7/7	14
18	F	43.0	1.7	Ischemic	Globus palladus, putamen, anterior limb of internal capsule, caudate nucleus, cortical F-P-T	1.2/1.0	7/7	15
19	M	58.0	1.4	Ischemic	Lateral medulla	2.0/1.6	6/6	15
20	M	57.0	0.6	Ischemic	Sylvian: corona radiate, internal capsule, subcortical center	1.9/1.2	7/7	15
21	M	40.8	4.0	Hemorrhagic	Basal ganglia and external capsule	1.6/1.2	6/6	14
22	M	68.2	8.8	Ischemic	MCA territory	1.4/1.1	6/6	15
23	M	51.5	1.3	Ischemic	Cerebellar, right lateral medullary	1.9/1.5	7/7	15
24	M	54.0	1.2	Ischemic	Internal capsule, globus palladus, lacunar corona radiata, F	1.9/1.5	7/7	15
25	M	75.5	0.8	Ischemic	F + MCA territory	1.5/1.1	7/7	15
26	M	46.7	1.1	Ischemic	Sylvian	1.1/0.9	7/7	15
27	M	52.1	2.3	Ischemic	Posterior limb of right internal capsule	2.3/1.3	7/7	15
28	M	73.8	0.7	Ischemic	Internal capsule	1.5/1.1	7/7	14
29	M	77.3	2.9	Ischemic	MCA territory	1.3/1.0	7/6	15
30	M	48.3	1.3	Hemorrhagic	Brainstem, periaqueductal	1.9/1.8	7/7	15
Mean ± SD Range (−) Ratio (:)	13:2	58.5 ± 13.2	2.0 ± 2.1	13:2	NA	1.6 ± 0.3 / 1.2 ± 0.2	6–7 / 6–7	14–15
HC (n = 15)								
Mean ± SD Range (−) Ratio (:)	7:8	61.0 ± 11.3	NA	NA	NA	2.1 ± 1.0 / 1.4 ± 0.3	NA	NA

*USN* Unilateral spatial neglect, *CMSA* Chedoke McMaster Stroke Assessment, *SD* Standard Deviation; *F* Frontal, *P* Parietal, *MCA* Middle cerebral artery, *T* Temporal, *O* Occipital, *Ins* Insular, 10 *MWT* 10-Meter Walk Test, *USN*+ Participants with post-stroke USN, *USN*- Participants without post-stroke USN, *HC* Healthy Controls, *N/A* Not applicable, *RMI* Rivermead Mobility Index

^a^refers to the use of walker during goal-directed locomotor experiments

*refers to significant between-group differences in USN+ vs. USN – group (*p* < 0.05)

^§^refers to significant between-group differences in USN+ vs. HC group (*p* < 0.05)

### USN characteristics

The USN+ group included five (*n* = 5) individuals with history of USN, and ten (*n* = 10) individuals with actual USN on testing (Table 2). All USN-related measures, both in the near and far space (*p* ≤ 0.05), demonstrated deficits in patients with post-stroke USN. By contrast, none of the USN- individuals scored positive in any of the USN assessments. The deviation percentages on the LBT (near space: 6.6 ± 4.9; far space: 6.6 ± 6.2) and laterality indices on the SCT (near space: 0.9 ± 0.11; far space: 0.9 ± 0.09) in the USN+ group were also found to be similar between the near and far space (*p* = 0.50 to 1.00). Participants with history of USN (S1-S5) were significantly different from USN- group only on two temporal measures (timing of SCT and APT) (*p* < 0.05).

**Table 2:**
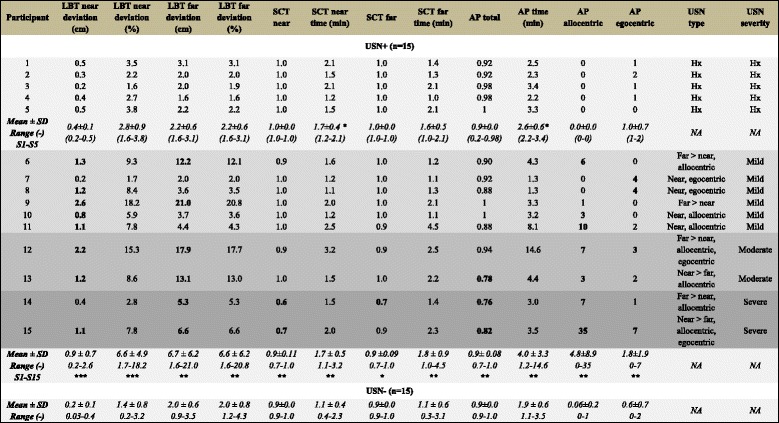
USN descriptive variables

*USN* Unilateral spatial neglect, *USN+* Participants with post-stroke USN, *USN-* Participants without post-stroke USN, *NA* Not applicable, *LBT* Line bisection test, *SCT* Star Cancellation Test, *AP* Apples Test, minutes (min); *Hx* History of USN, *SD* Standard deviation, Numbers in bold correspond to values above or below (where applicable) cut-off values

*, **, *** *p*-value < 0.05, 0.01, 0.001, respectively between USN+ and USN- group; USN severity is delineated by shades ranging from light (those with history of USN) to dark grey (those with severe USN)

When considering individuals’ scores on USN-related measures in the 10 participants with actual neglect, however, some variability in the expression of USN was observed. Four (*n* = 4) participants had more severe far- than near-space USN, two (*n* = 2) had more severe near- than far-space USN, and four (*n* = 4) had only near-space USN. Allocentric (object-centered) USN was more common and found in 7 out of 10 participants. Egocentric (viewer-centered) USN was found in 2 out of 10 individuals, and 2 participants presented with both allocentric and egocentric USN. Mild, moderate and severe USN was present in six (*n* = 6/10), two (*n* = 2/10), and two (n = 2/10) participants, respectively.

### Goal-directed navigation and detection

**Fig. 2:**
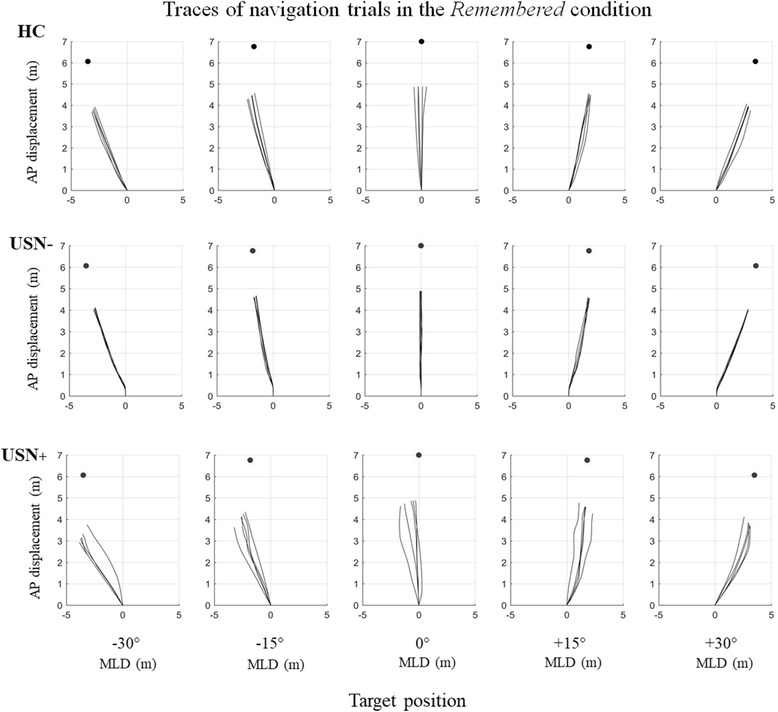
Displacement traces. Birds eye view of mediolateral displacement (MLD), as performed by one HC participant, one individual without post-stroke neglect (USN-) and one individual with post-stroke neglect (USN+) for the 5 target positions during the remembered condition. The anterior-posterior (AP) displacement is on the y-axis. Target position is shown with the black dot

Figure 2 depicts typical MLD traces during the goal-directed navigation task performance of a USN- and a USN+ participant with stroke in the *remembered* condition. It can be observed that the USN- participant’s MLD was tightly modulated as a function of the remembered target location, leading to small errors approximating 0° (MLD errors from − 30° to + 30° are: 0.40, 0.38, 0.03, 0.53, 0.50 m). A similar behaviour was observed in a HC participant (MLD errors from − 30° to + 30° are: 0.82, 0.91, 0.14, 0.67, 0.62 m). By contrast, the USN+ participant demonstrated a large variability in performance, with larger deviations in the navigation trajectories to all targets which led to larger MLD errors, especially for the left-sided target (− 30° and − 15°) (MLD errors from − 30° to + 30° are: 2.73, 1.99, 1.04, 0.91, 1.53 m). It emerges that the USN+ participant overshot most left-sided targets to arrive on their left side at the end of the trial.

**Fig. 3:**
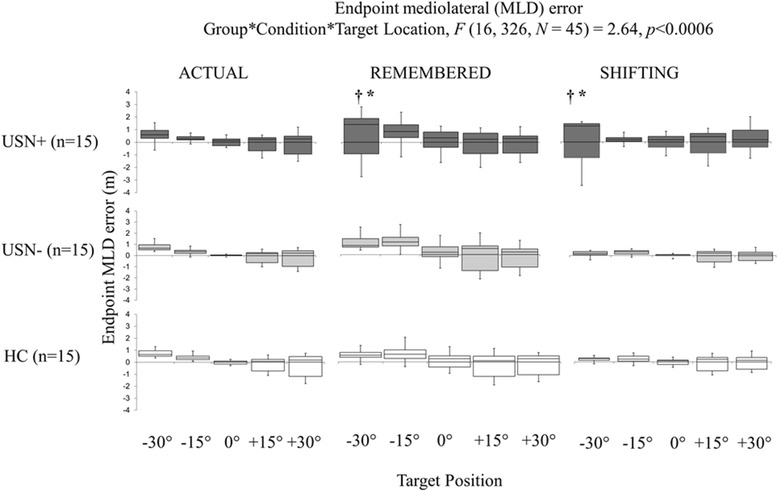
Mediolateral displacement endpoint error results. Mediolateral displacement (MLD) endpoint error results for the 3 study groups (USN+, USN-, HC ranging from dark grey to white, respectively) in actual, remembered and shifting conditions to the five target locations. Box and whiskers description: minimal and maximal values shown by the whiskers, the bottom and top of the box are the first and third quartiles, and the band inside the box is the second quartile (the median). Significant between-group differences at *p*-value < 0.05 are indicated for USN+ vs. USN- groups (†) and USN+ vs. HC groups (*), respectively. Negative and positive values symbolize, respectively, endpoint positions that undershoot (to the right of LEFT targets or left of RIGHT target) and overshoot the target (to the left of LEFT target or right of RIGHT target)

Mean MLD errors and the direction of deviation for the 3 study groups during the joystick navigation task performance are shown in Fig. 3. A significant 3-way interaction of Group x Condition x Target Location was found (*F* (16, 326, *N* = 45) = 2.64, *p* < 0.0006). Within the USN+ group, worse performance was noted for the left and right most eccentric targets (±30°) in the shifting vs. actual condition (*p* < 0.05); and for the left target (− 15°) and right eccentric target (+ 30°) in the remembered vs. shift condition (*p* < 0.01). Subsequent pairwise comparisons showed that USN+ demonstrated greater MLD errors only for the left most eccentric target (− 30°) in the remembered (mean ± SD: 1.12 ± 0.72 m) and shifting (mean ± SD:1.10 ± 1.49 m) conditions compared to the USN- (mean ± SD: 0.96 ± 0.57; 0.48 ± 0.61, effect size: *d* = 0.24 and 0.54, respectively) and HC groups (mean ± SD: 0.63 ± 0.24; 0.27 ± 0.13, effect size: *d* = 0.91; 0.78, respectively) (*p* < 0.05). No significant between-group differences were found for USN- vs. HC groups. It can also be noted that most of the USN+ individuals overshot the left-sided targets in the actual and remembered conditions, ending their navigation trial to the left of the target. For the middle and right sided targets, no such delineated performance was observed, where some participants overshot and others undershot the target at endpoint.

**Fig. 4:**
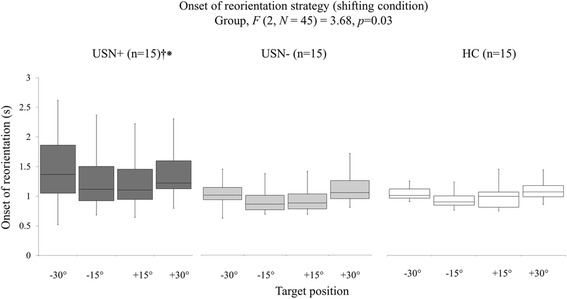
Onset of reorientation results. Onset of reorientation results for the 3 study groups (USN+, USN-, HC ranging from dark grey to white, respectively) to the 4 target locations. Box and whiskers description: minimal and maximal values shown by the whiskers, the bottom and top of the box are the first and third quartiles, and the band inside the box is the second quartile (the median). Significant between-group differences at *p*-value < 0.05 are indicated for USN+ vs. USN- groups (†) and USN+ vs. HC groups (*), respectively, next to USN+ group description (given that only a group effect was found to be significant)

A comparison of onset times of reorientation strategy across the 3 groups for the shifting target condition (Fig. 4) revealed a significant Group effect (*F* (2, *N* = 45) = 3.68, *p* = 0.03). Pairwise comparisons further indicated that the USN+ group presented with longer onsets of reorientation strategies compared to the USN- and HC groups for all target locations (*p* < 0.05). No significant differences were found between USN- vs. HC groups.

**Fig. 5:**
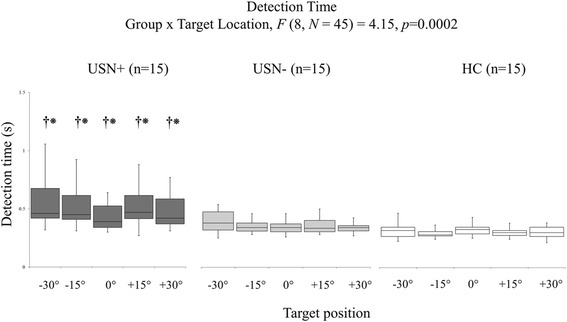
Detection time results. Detection time results for the 3 study groups (USN+, USN-, HC ranging from dark grey to white, respectively) to the five target locations. Box and whiskers description: minimal and maximal values shown by the whiskers, the bottom and top of the box are the first and third quartiles, and the band inside the box is the second quartile (the median). Significant between-group differences at *p*-value < 0.05 are indicated for USN+ vs. USN- groups (†) and USN+ vs. HC groups (*), respectively

As illustrated in Fig. 5, a significant 2-way interaction of Group x Target Location was also found for the detection time on the target detection task (*F* (8, *N* = 45) = 4.15, *p* = 0.0002). The USN+ group showed longer detection times for the left target (− 30°) compared to the right target (+ 30°), as well as longer detection times for left and right targets (±15°/±30°) compared to the middle target (0°) (*p* < 0.05). The USN+ group further demonstrated longer detection times for all target locations (detection times from − 30° to + 30° are: 0.47 ± 0.15; 0.52 ± 0.17; 0.43 ± 0.11; 0.50 ± 0.17; 0.57 ± 0.23 s) compared to USN- (detection times from − 30° to + 30° are: 0.35 ± 0.11; 0.35 ± 0.10; 0.34 ± 0.11; 0.36 ± 0.12; 0.38 ± 014 s) and HC (detection times from − 30° to + 30° are: 0.32 ± 0.05; 0.31 ± 0.04; 0.35 ± 0.07; 0.33 ± 0.06; 0.34 ± 0.07 s) groups (*p* < 0.05), with the latter two groups showed no significant differences.

### Relationship and deficits’ detection ability analyses

Relationship and deficits’ detection ability analyses were performed between USN clinical tests and goal-directed navigation performances (MLD error and onset of reorientation outcomes) to the left eccentric target (− 30°) in the remembered and shifting conditions, as these specific conditions showed the largest alteration in the USN+ individuals. No significant correlations were found between any USN clinical tests and detection time outcome with the MLD error in the remembered and shifting condition (*p* > 0.05). Significant, but negligible to low magnitude, correlations were found between the onset time of reorientation strategy and scores on the LBT/SCT in near space (*r* = 0.29/*r* = − 0.33) and LBT in far space (*r* = 0.34) (*p* < 0.05), indicating that a poor performance on USN tests was somewhat associated with a poor performance on the goal-directed navigation task in the shifting condition. No significant correlations were found between the detection time outcome and the onset time of reorientation strategy outcome (*p* > 0.05).

Further, single case analyses were performed (Table 3) for two outcomes that were found to be significantly affected in USN+ vs. other two study groups: MLD error in the remembered and shifting conditions. The navigation task in these conditions allowed the detection of significantly higher MLD errors vs. the normative sample (HC group) in 3 out of 5 individuals with history of USN (S2, S4, S5) (*p* < 0.05) and in 4 (S16, S17, S22, S24) out of 15 USN- (*p* < 0.05). In addition, significantly higher MLD errors vs. the normative sample (USN- group) were found in 3 out of 5 individuals with history of USN (S1, S2, S5) (*p* < 0.05). Further, it is important to note that most participants with history of USN displayed statistically similar performances in comparison to those with actual USN on testing.

**Table 3:**
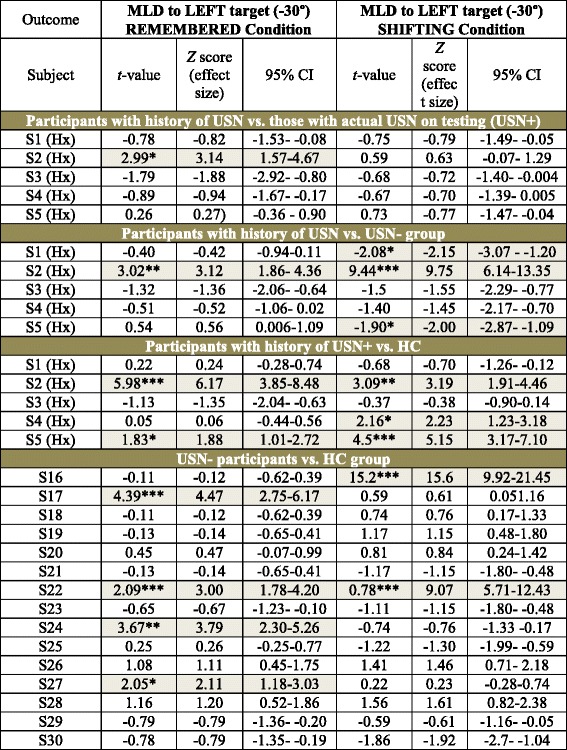
Single Case Analyses

Single-case analysis of participants with history of USN vs. those with actual USN on testing, vs. USN- group, and vs. HC group (as control data); and single USN- participants vs. HC group (as control data) on the performance of navigation task in the *Remembered* and *Shifting* conditions to the left target at −30°. History (Hx); Subject (S); Subjects with post-stroke USN (USN+); Subjects post-stroke without USN (USN-); Healthy controls (HC); Unilateral spatial neglect (USN); Light grey selections represent individual cases whose performance is considerably worse than the comparison group (*p* < 0.05); Star symbols indicate *p*-value < 0.05 (*), < 0.01 (**) and < 0.001(***)

## Discussion

The present study investigated the effects of post-stroke USN on goal-directed navigation and detection abilities. We employed a joystick-driven navigation task, thereby eliminating the potential confounding effects of gait-related abilities which normally differ between individuals with vs. without USN [9, 13, 14]. Furthermore, such a task could be easier to implement in the clinical setting, as it necessitates a fairly easy setup within a compact area and requires a short administration time approximating 15 min. This is of particular importance for patients with post-stroke USN, as they are known to have a decreased sustained attention and alertness over longer periods of time [42, 43].

Overall, USN-specific deficits in space navigation and object detection were identified and are in accordance with prior research, suggesting that a joystick-driven task may be reflective of actual perceptual motor abilities in neglect. Recently, Aravind et al. [16] showed USN-specific deficits in a joystick navigation-based obstacle avoidance and obstacle detection task. Congruently, the results of the present study demonstrate that individuals with post-stroke USN, as they performed the joystick task, showed greater endpoint mediolateral errors in the remembered condition. Moreover, the present study provides evidence for USN deficits in representation updating during navigation, where affected individuals showed altered behavior in their ability to detect and adapt to changes in far-space target locations (i.e. shifting condition). Collectively, these findings propose that post-stroke USN affects space navigation, even in the absence of greater sensorimotor demands otherwise present in locomotion. Another interesting finding is that during navigation, individuals predominantly overshot left-sided targets, showing a left-side navigation deviation. This result is contrary to findings in previously conducted locomotor experiments, where individuals with post-stroke USN mainly presented with rightward walking trajectory deviations (i.e. to the “non-neglected” side) [9, 14]. We hypothesize that factors such as the differences in the mode of displacement in the walking vs. the joystick navigation tasks as well as the influence of walking dysfunctions could explain this discrepancy. To clarify, the joystick mode of control allowed the participant to make trajectory adjustments that were not limited in magnitude and not restricted by one’s walking capacity, as opposed to what was experienced during locomotion. For this reason, participants with USN, who also presented with a reduced walking ability, may have undershot the left (neglected) target in the walking task, while they overshot that same target during joystick navigation. In relation to that, Huitema et al. [9], in an experiment that involved walking to a centrally located target, reported that USN+ individuals who were also slow walkers (*n* = 3) deviated to the ipsilesional/right side, whereas fast walkers (*n* = 3) deviated to the contralesional/left side. The authors concluded that walking trajectory deviation in individuals with USN may have depended on their walking ability. The joystick-driven task may thus reflect more accurately the perceptual-motor abilities of individuals with post-stroke USN, but falls short in estimating the impact of USN on actual locomotion. Another difference between joystick navigation and walking is that the former did not allow bodily horizontal rotation, such that the only way to align with a peripheral target is to terminate the trial while being in front of it. In contrast, locomotor steering is achieved through both body displacement and reorientation [44], such that while the endpoint trajectory ‘apparently undershoots’ the target in terms of MLD, participants may still be ‘on target’ when considering their body orientation with respect to that target [45].

Furthermore, our team previously conducted a systematic review examining the effects of post-stroke USN on goal-directed upper extremity movements performed within the near-space [46]. The findings were consistent with the hypothesis that there are two different types of action control, processed via distinct visual streams, ventral and dorsal [47]. More precisely, impairments in upper extremity movements among individuals with post-stroke USN were found mostly during perceptual, memory-guided or delayed tasks (e.g. delayed pointing to remembered target location – ventral stream processing/offline movements); but not in immediate tasks (e.g. pointing to an actual target – dorsal stream processing/online movements). We propose that the conditions used in the present experiment could tap into ventral vs. dorsal stream hypothesis such that: the actual condition is visually-guided/online as it requires a quick response with the joystick; the remembered condition is memory-guided/offline; and the shifting condition is visually-guided, but since it also necessitates representational updating, it could be party an offline type. On one hand, what we found is that the proposed ventral vs. dorsal stream hypothesis does potentially hold for far-space exploration in navigation, given that significant between-group differences were shown in both conditions with offline components (i.e. remembered and shifting), but not in the actual/online condition. On the other hand, we speculate that this hypothesis does not entirely hold for far-space exploration in locomotion, given that significant between-group differences were shown in both, actual and remembered conditions. We argue that as opposed to joystick navigation in far space, far space exploration requiring goal-directed locomotion entails additional space computation and re-adjustments of self with respect to target location [48] and perceived optic flow direction [49] to transform perception into action. It is also a longer task to perform as opposed to joystick navigation, and is more physically demanding. As a result, the actual condition in the locomotor experiment is potentially no longer a clear-cut “online” condition, but rather incorporates both, online and offline components. These additional demands on the visual-perceptual, attention, and sensorimotor systems during walking vs. navigation could account for these differences in findings between the two experiments. Overall, rather than fully supporting the ventral stream hypothesis in USN proposed by Milner and Goodale [50], our results of the locomotion vs. navigation experiment align with the model proposed by Rizzollati and Matelli [51] who suggested that a system encompassing both ventral and dorsal streams is underlying USN, such that action control in the dorsal system is affected by the disruption of visuospatial information from ventral regions (temporal-parietal).

### Lateralized and non-lateralized USN deficits found in far-space object detection

The detection task in the present experiment identified USN-related deficits in the detection time of left-sided (i.e. contralesional) targets. This is in accord with previous studies on contralesionally located object detection in post-stroke USN individuals [16, 35, 52, 53]. In fact, an ample body of research focused on lateralized spatial USN deficits occurring in the neglected/contralesional hemispace. To a large extent, the attentional theory of USN, namely its hemispheric imbalance model [53, 54], global/local processing model [55–58] and the disengagement deficit/attentional shift model [59–61]), could provide explanations into this type of impairment and support our findings. The attentional theory of USN overall proposes that the right brain hemisphere plays the key role in directing attention to the left visual hemispace. It further suggests that lateralized left USN deficits are observed following the right hemisphere lesion and: 1. ensuing lack of orientation to left hemispace due to hypoactive right hemisphere (i.e. hemispheric imbalance model [53, 54]); or 2. inability to direct global/local attention (global/local processing model [55–58]); or 3. inability to disengage attention from the right visual hemispace and shift attention to the left visual hemispace (disengagement deficit model [59–61]). Interestingly, through the detection task, we also identified deficits that were non-lateralized, where performances were not solely worse in the neglected hemifield, but also in the ipsilesional/“non-affected” hemispace. Previous studies have also reported presence of non-lateralized deficits in individuals with post-stroke USN [62, 63]. Functional imaging studies reported the intraparietal sulcus and the frontal cortex to play a role in non-lateralized visual processing [64, 65]. In relation to that, our sample of individuals with post-stroke USN was constituted of nearly 70% of those with parietal and/or frontal lesions [13] further indicating that the presence of non-lateralized deficits could be accounted for by the lesion areas involved.

### Clinical USN measures are not reflective of navigation abilities, but the navigation task could detect dormant deficits

Traditionally, post-stroke USN is assessed using paper-and-pencil tests, such as those used in the present study (e.g. Line Bisection, Star Cancellation, Apples Tests). In our participants, the results on these tests did not show any significant associations with the outcomes of MLD error in the remembered and shifting conditions; and only negligible to low magnitude associations with the outcome of onset of reorientation in shifting condition. This lack of significant associations could be explained by the differences in the types of stimuli (e.g. static vs. dynamic) and the nature of the tasks (cancellation and bisection vs. navigation). Moreover, despite the convenience of paper-and-pencil tests, their easy application and scoring, most of them are designed to assess USN of near-extrapersonal space only, and do not address essential everyday activities within the far-extrapersonal space. In fact, studies have reported participants with recovered USN based on conventional paper and pencil tests showing residual altered walking trajectory [66] and goal-directed reaching impairments [67]. Also, recent studies identified patients having mild USN, or no USN on paper-and-pencil tests but showing difficulty and USN on a VR task involving more challenging and dynamic type of tasks within an ecological 3-D environment [15, 32, 68]. These findings further demonstrate the lack of conventional evaluation tools’ sensitivity and help explain the negligible or low associations found in the present study. Compared to paper-and-pencil tests, our navigation task likely involved more complex processes of representational updating, spatial memory, and readjustments calculations/adaptability of self as one approaches the far-space target. Those skills and abilities are not accounted for in the paper-and-pencil tests, possibly leading to the lack of association. Therefore, our results confirm that conventional methods of USN assessment are not sufficient and sensitive enough to detect functional impairments in daily activities, such as goal-directed locomotion and/or navigation.

In addition, the performance to the left target (− 30°) in the remembered and shifting conditions allowed detecting deficits in 3 out of 5 participants with “recovered” USN as per the conventional paper-and-pencil tests (i.e. having a history of USN) and in 4 individuals of the USN- group, supporting the observation that conventional USN assessment tools may fail to predict performance in visually-guided functional tasks [12, 15, 68, 69]. This finding suggests that although individuals with “recovered” (or undiagnosed) USN may perform within the norms in static, 2-D environments, perhaps their compensatory strategies fall short and deficits emerge when these individuals are exposed to a moving 3-D environment and performing a more complex activity. Our results warrant for the development of novel, more sensitive evaluation methods that could potentially incorporates the use of VR and tasks requiring navigation.

### Limitations

Only participants with left USN (i.e. right hemisphere stroke) were included, limiting the generalizability of results to the left hemisphere stroke population. In addition, a more detailed description of participants’ lesional patterns would have been informative and valuable in explaining the observed findings. Further, the present study did not examine eye movements during the experiments that could offer valuable information on gaze shifts, spatial fixations and re-fixations, possible remapping and gaze shifting impairments underlying USN. Another limitation is that the task design did not allow rotations along the vertical axis to mimic head and body horizontal rotation strategies as used in goal-directed locomotion [44]; however, the task was designed this way, so as not to add extra complexities to the control interface. Moreover, the motor function and recovery of the non-paretic upper extremity used in the joystick experiment was not objectively evaluated in the present study using standardized measures and one could argue that a unilateral hemisphere stroke could possibly result in impairments of bilateral upper extremities, but to different degrees: “more vs. less affected”. Nevertheless, we believe that the paradigm used in this study allowed, to the best possible, to minimize the contribution of post-stroke sensorimotor impairments. Further, no significant differences between USN- and HC participants were observed in any of the outcome measures, suggesting that sensorimotor deficits of the non-paretic upper extremity, if any, did not affect the joystick task performance. Lastly, given the challenge of disentangling visual neglect from visual field deficits in individuals with post-stroke USN, the Goldmann perimetry or computerized equivalent tests results were often absent from medical charts of these participants. This could have resulted in the inclusion of participants with concurrent visual field deficits and USN in the USN+ group.

## Conclusions

The present study demonstrated complementary evidence to a previously conducted goal-directed locomotion experiment. We identified that even in the absence of biomechanical demands of locomotion, goal-directed navigation is affected in a memory-guided condition and in a task requiring representational updating component in individuals with USN. In addition, lateralized and non-lateralized deficits were shown using the detection task in the individuals with USN. These deficits were not associated with observed performances with clinical USN measures; however, the navigation task was sensitive in detecting deficits otherwise left undetected by conventional assessments. While the joystick navigation show similarities with goal-directed walking in terms of the task itself and observed findings in individuals with USN, discrepancies were also identified in the side of the endpoint mediolateral deviations, and alterations found in the actual condition during locomotion, but not during navigation, which may be explained by factors such as the mode of displacement and the influence of walking ability (or lack of thereof). Taken together, these findings present preliminary steps towards the development of more sensitive evaluation tools and treatment approaches that incorporate VR-based navigation and object detection, and that account for lateralized and non-lateralized deficits, representational updating and spatial memory.

## Additional file

Additional file 1: Calculations related to onset of reorientation strategy. (DOCX 369 kb)

## Abbreviations

USN: Unilateral spatial neglect
USN +: Patients with post-stroke unilateral spatial neglect.
USN-: Patients post-stroke without unilateral spatial neglect
HC: Healthy controls
CT: Computer tomography
LBT: Line Bisection Test
SCT: Star Cancellation Test
APT: Apples Test
CRIR: Centre for Interdisciplinary Research in Rehabilitation of Greater Montreal
VR: Virtual reality
VE: Virtual environment
MLD: Mediolateral displacement

## References

[1] Barrett AM, Buxbaum LJ, Coslett HB, Edwards E, Heilman KM, Hillis AE, Milberg WP, Robertson IH (2006). Cognitive rehabilitation interventions for neglect and related disorders: moving from bench to bedside in stroke patients. J Cogn Neurosci.

[2] Buxbaum LJ, Ferraro MK, Veramonti T, Farne A, Whyte J, Ladavas E, Frassinetti F, Coslett HB (2004). Hemispatial neglect: subtypes, neuroanatomy, and disability. Neurology.

[3] Heilman KM, Valenstein E, Watson RT (2000). Neglect and related disorders. Semin Neurol.

[4] Ting DS, Pollock A, Dutton GN, Doubal FN, Ting DS, Thompson M, Dhillon B (2011). Visual neglect following stroke: current concepts and future focus. Surv Ophthalmol.

[5] Franceschini M, La Porta F, Agosti M, Massucci M, Group ICR (2010). Is health-related-quality of life of stroke patients influenced by neurological impairments at one year after stroke?. Eur J Phys Rehabil Med.

[6] Stav WB, Pierce S, Wheatley CJ, Davis ES (2005). Commission on P. Driving and community mobility. Am J Occup Ther.

[7] Lord SE, McPherson K, McNaughton HK, Rochester L, Weatherall M (2004). Community ambulation after stroke: how important and obtainable is it and what measures appear predictive?. Arch Phys Med Rehabil.

[8] Berti A, Smania N, Rabuffetti M, Ferrarin M, Spinazzola L, D'Amico A, Ongaro E, Allport A (2002). Coding of far and near space during walking in neglect patients. Neuropsychology.

[9] Huitema RB, Brouwer WH, Hof AL, Dekker R, Mulder T, Postema K (2006). Walking trajectory in neglect patients. Gait Posture.

[10] Robertson IH, Tegner R, Goodrich SJ, Wilson C (1994). Walking trajectory and hand movements in unilateral left neglect: a vestibular hypothesis. Neuropsychologia.

[11] Tromp E, Dinkla A, Mulder T (1995). Walking through doorways: an analysis of navigation skills in patients with neglect. Neuropsychol Rehab.

[12] Aravind G, Lamontagne A (2014). Perceptual and locomotor factors affect obstacle avoidance in persons with visuospatial neglect. J Neuroeng Rehabil.

[13] Ogourtsova T, Archambault SP, Lamontagne A. Post-stroke unilateral spatial neglect affects both offline and online goal-directed walking. Submitted to restorative neurology and neuroscience in July 2017.

[14] Turton AJ, Dewar SJ, Lievesley A, O'Leary K, Gabb J, Gilchrist ID (2009). Walking and wheelchair navigation in patients with left visual neglect. Neuropsychol Rehabil.

[15] Buxbaum LJ, Palermo MA, Mastrogiovanni D, Read MS, Rosenberg-Pitonyak E, Rizzo AA, Coslett HB (2008). Assessment of spatial attention and neglect with a virtual wheelchair navigation task. J Clin Exp Neuropsychol.

[16] Aravind G, Darekar A, Fung J, Lamontagne A (2015). Virtual reality-based navigation task to reveal obstacle avoidance performance in individuals with visuospatial neglect. IEEE Trans Neural Syst Rehabil Eng.

[17] Schenkenberg T, Bradford DC, Ajax ET (1980). Line bisection and unilateral visual neglect in patients with neurologic impairment. Neurology.

[18] Wilson B, Cockburn J, Halligan P (1987). Development of a behavioral test of visuospatial neglect. Arch Phys Med Rehabil.

[19] Bickerton WL, Samson D, Williamson J, Humphreys GW (2011). Separating forms of neglect using the apples test: validation and functional prediction in chronic and acute stroke. Neuropsychology.

[20] Oldfield RC (1971). The assessment and analysis of handedness: the Edinburgh inventory. Neuropsychologia.

[21] Bailey IL, Lovie JE (1976). New design principles for visual acuity letter charts. Am J Optom Physiol Optic.

[22] Nasreddine ZS, Phillips NA, Bedirian V, Charbonneau S, Whitehead V, Collin I, Cummings JL, Chertkow H (2005). The Montreal cognitive assessment, MoCA: a brief screening tool for mild cognitive impairment. J Am Geriatr Soc.

[23] Wolf SL, Catlin PA, Gage K, Gurucharri K, Robertson R, Stephen K (1999). Establishing the reliability and validity of measurements of walking time using the Emory functional ambulation profile. Phys Ther.

[24] Bohannon RW (1997). Comfortable and maximum walking speed of adults aged 20-79 years: reference values and determinants. Age Ageing.

[25] Bohannon RW, Andrews AW, Thomas MW (1996). Walking speed: reference values and correlates for older adults. J Orthop Sports Phys Ther.

[26] Franchignoni F, Tesio L, Benevolo E, Ottonello M (2003). Psychometric properties of the Rivermead mobility index in Italian stroke rehabilitation inpatients. Clin Rehabil.

[27] Gowland C, Stratford P, Ward M, Moreland J, Torresin W, Van Hullenaar S, Sanford J, Barreca S, Vanspall B, Plews N (1993). Measuring physical impairment and disability with the Chedoke-McMaster stroke assessment. Stroke.

[28] Line Bisection Test [https://www.strokengine.ca/en/assess/lbt/]. Accessed 3 Apr 2018.

[29] Star Cancellation Test [https://www.strokengine.ca/en/assess/sct/]. Accessed 3 Apr 2018.

[30] Aimola L, Schindler I, Simone AM, Venneri A (2012). Near and far space neglect: task sensitivity and anatomical substrates. Neuropsychologia.

[31] Lindell AB, Jalas MJ, Tenovuo O, Brunila T, Voeten MJ, Hamalainen H (2007). Clinical assessment of hemispatial neglect: evaluation of different measures and dimensions. Clin Neuropsychol.

[32] Buxbaum LJ, Dawson AM, Linsley D (2012). Reliability and validity of the virtual reality lateralized attention test in assessing hemispatial neglect in right-hemisphere stroke. Neuropsychology.

[33] Aravind G, Lamontagne A (2017). Dual tasking negatively impacts obstacle avoidance abilities in post-stroke individuals with visuospatial neglect: task complexity matters!. Restor Neurol Neurosci.

[34] Aravind G, Darekar A, Fung J, Lamontagne A (2015). ViVirtual reality-based navigation task to reveal obstacle avoidance performance in individuals with visuvisuospatial neglect. IEEE Trans Neural Syst Rehab Eng.

[35] Dvorkin AY, Bogey RA, Harvey RL, Patton JL (2012). Mapping the neglected space: gradients of detection revealed by virtual reality. Neurorehabil Neural Repair.

[36] Armbruster C, Wolter M, Kuhlen T, Spijkers W, Fimm B (2008). Depth perception in virtual reality: distance estimations in peri- and extrapersonal space. Cyberpsychol Behav.

[37] Mon-Williams M, Bingham GP (2008). Ontological issues in distance perception: cue use under full cue conditions cannot be inferred from use under controlled conditions. Percept Psychophys.

[38] Magdalon EC, Michaelsen SM, Quevedo AA, Levin MF (2011). Comparison of grasping movements made by healthy subjects in a 3-dimensional immersive virtual versus physical environment. Acta Psychol.

[39] Veerman MM, Brenner E, Smeets JB (2008). The latency for correcting a movement depends on the visual attribute that defines the target. Exp Brain Res.

[40] Hinkle DE, Wiersma W, Jurs SG (2003). Applied statistics for the behavioral sciences.

[41] Crawford JR, Garthwaite PH (2002). Investigation of the single case in neuropsychology: confidence limits on the abnormality of test scores and test score differences. Neuropsychologia.

[42] Heilman KM, Schwartz HD, Watson RT (1978). Hypoarousal in patients with the neglect syndrome and emotional indifference. Neurology.

[43] Malhotra P, Coulthard EJ, Husain M (2009). Role of right posterior parietal cortex in maintaining attention to spatial locations over time. Brain.

[44] Patla AE, Adkin A, Ballard T (1999). Online steering: coordination and control of body center of mass, head and body reorientation. Exp Brain Res.

[45] Garcia-Popov A, Lamotagne A. The effect of differing optic flow on steering behaviours during goal-oriented locomotion. In: 2011 international conference on virtual rehabilitation. Zurich: IEEE. p. 2011.

[46] Ogourtsova T, Archambault P, Lamontagne A (2015). The impact of post-stroke unilateral spatial neglect on goal-directed arm movements: systematic literature review. Top Stroke Rehabil.

[47] Milner AD, Goodale MA (1995). The visual brain in action.

[48] Rushton SK, Harris JM, Lloyd MR, Wann JP (1998). Guidance of locomotion on foot uses perceived target location rather than optic flow. Curr Biol.

[49] Warren WH, Kay BA, Zosh WD, Duchon AP, Sahuc S (2001). Optic flow is used to control human walking. Nat Neurosci.

[50] Milner AD, Goodale MA (2006). The visual brain in action.

[51] Rizzollati GM (2003). M. Two different stream from the dorsal visual system: anatomy and functions. Exp Brain Res.

[52] Kaizer F, Korner-Bitensky N, Mayo N, Becker R, Coopersmith H (1988). Response time of stroke patients to a visual stimulus. Stroke.

[53] Smania N, Martini MC, Gambina G, Tomelleri G, Palamara A, Natale E, Marzi CA (1998). The spatial distribution of visual attention in hemineglect and extinction patients. Brain.

[54] Kinsbourne M, IHM R, J. C (1993). Orinetational bias model of unilateral neglect: evidence from attentional gradients within hemispace. Uniateral neglect: Clinical and experimental studies.

[55] Marshall JC, Halligan PW (1995). Seeing the forest but only half the trees?. Nature.

[56] Doricchi F, Incoccia C (1998). Seeing only the right half of the forest but cutting down all the trees?. Nature.

[57] Marshall JC, Halligan PW (1994). The yin and the Yang of visuo-spatial neglect: a case study. Neuropsychologia.

[58] Halligan PW, Marshall JC (1994). Focal and global attention modulate the expression of visuo-spatial neglect: a case study. Neuropsychologia.

[59] Posner MI, Walker JA, Friedrich FJ, Rafal RD (1984). Effects of parietal injury on covert orienting of attention. J Neurosci.

[60] Morrow LA, Ratcliff G (1988). The disengagement of covert attention and the neglect syndrome. Psychobiology.

[61] Eglin M, Robertson LC, Knight RT (1989). Visual search performance in the neglect syndrome. J Cogn Neurosci.

[62] Duncan J, Bundesen C, Olson A, Humphreys G, Chavda S, Shibuya H (1999). Systematic analysis of deficits in visual attention. J Exp Psychol Gen.

[63] Battelli L, Cavanagh P, Intriligator J, Tramo MJ, Henaff MA, Michel F, Barton JJ (2001). Unilateral right parietal damage leads to bilateral deficit for high-level motion. Neuron.

[64] Coull JT, Frith CD (1998). Differential activation of right superior parietal cortex and intraparietal sulcus by spatial and nonspatial attention. NeuroImage.

[65] Coull JT, Nobre AC (1998). Where and when to pay attention: the neural systems for directing attention to spatial locations and to time intervals as revealed by both PET and fMRI. J Neurosci.

[66] Goodale MA, Milner AD, Jakobson LS, Carey DP (1990). Kinematic analysis of limb movements in neuropsychological research: subtle deficits and recovery of function. Can J Psychol.

[67] Mattingley JB, Phillips JG, Bradshaw JL (1994). Impairments of movement execution in unilateral neglect: a kinematic analysis of directional bradykinesia. Neuropsychologia.

[68] Peskine A, Rosso C, Box N, Galland A, Caron E, Rautureau G, Jouvent R, Pradat-Diehl P (2011). Virtual reality assessment for visuospatial neglect: importance of a dynamic task. J Neurol Neurosurg Psychiatry.

[69] Berard J, Fung J, Lamontagne A. Visuomotor control post stroke can be affected by a history of visuospatial neglect. J Neurol Neurophysiol. 2012;S8. https://www.omicsonline.org/visuomotor-control-post-stroke-can-be-affected-by-a-history-of-visuospatial-neglect-2155-9562.S8-001.php?aid=5205

