# Supplementary material for: Post-stroke unilateral spatial neglect: virtual reality-based navigation and detection tasks reveal lateralized and non-lateralized deficits in tasks of varying perceptual and cognitive demands
Source: J Neuroeng Rehabil. 2018 Apr 23;15:34. doi: 10.1186/s12984-018-0374-y (PMC5913876; doi:10.1186/s12984-018-0374-y)
Supplement: Supplementary file 1 — Calculations related to onset of reorientation strategy. (DOCX 369 kb) [file 12984_2018_374_MOESM1_ESM.docx]

# **Additional file 1:** Calculations related to onset of reorientation strategy


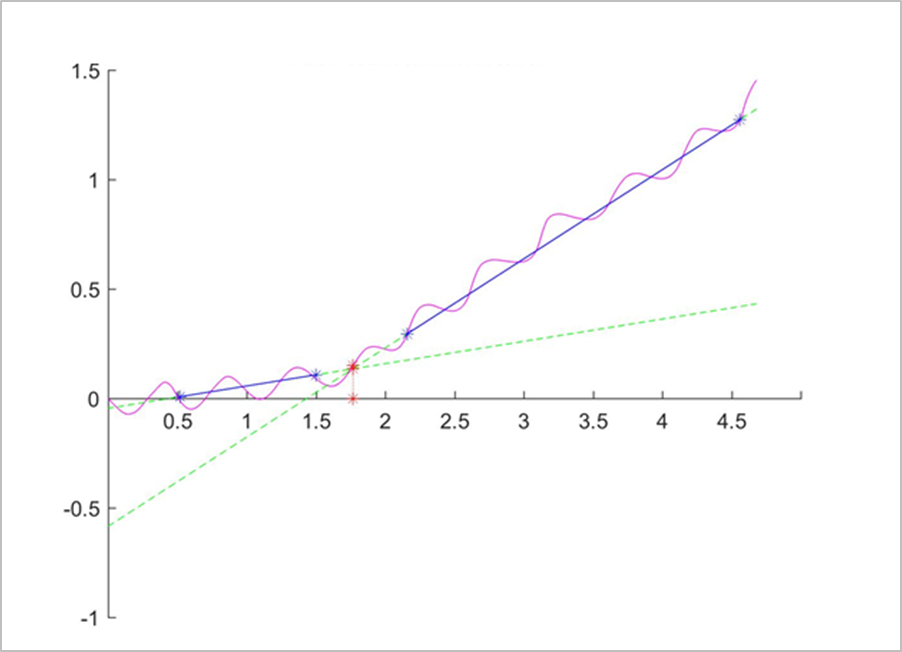


Mediolateral axis (m)

Anterior-posterior axis (m)

Anterior-posterior axis (m)

Anterior-posterior axis (m)

Anterior-posterior axis (m)

Anterior-posterior axis (m)

Anterior-posterior axis (m)

Anterior-posterior axis (m)

Anterior-posterior axis (m)

Anterior-posterior axis (m)

Anterior-posterior axis (m)

Anterior-posterior axis (m)

Anterior-posterior axis (m)

Anterior-posterior axis (m)

Anterior-posterior axis (m)

Anterior-posterior axis (m)

Anterior-posterior axis (m)

Anterior-posterior axis (m)

Anterior-posterior axis (m)

Anterior-posterior axis (m)

Anterior-posterior axis (m)

Anterior-posterior axis (m)

Anterior-posterior axis (m)

Anterior-posterior axis (m)

Anterior-posterior axis (m)

Anterior-posterior axis (m)

Anterior-posterior axis (m)

Anterior-posterior axis (m)

Anterior-posterior axis (m)

Anterior-posterior axis (m)

Anterior-posterior axis (m)

Anterior-posterior axis (m)

Anterior-posterior axis (m)

Anterior-posterior axis (m)

Anterior-posterior axis (m)

Anterior-posterior axis (m)

Anterior-posterior axis (m)

Anterior-posterior axis (m)

Anterior-posterior axis (m)

Anterior-posterior axis (m)

Anterior-posterior axis (m)

Anterior-posterior axis (m)

Anterior-posterior axis (m)

Anterior-posterior axis (m)

Anterior-posterior axis (m)

Anterior-posterior axis (m)

Anterior-posterior axis (m)

Anterior-posterior axis (m)

Anterior-posterior axis (m)

Anterior-posterior axis (m)

Anterior-posterior axis (m)

Anterior-posterior axis (m)

Anterior-posterior axis (m)

Anterior-posterior axis (m)

Anterior-posterior axis (m)

Anterior-posterior axis (m)

Anterior-posterior axis (m)

Anterior-posterior axis (m)

Anterior-posterior axis (m)

Anterior-posterior axis (m)

Anterior-posterior axis (m)

Anterior-posterior axis (m)

Anterior-posterior axis (m)

Anterior-posterior axis (m)

Anterior-posterior axis (m)

Anterior-posterior axis (m)

Anterior-posterior axis (m)

Anterior-posterior axis (m)

Anterior-posterior axis (m)

Anterior-posterior axis (m)

Anterior-posterior axis (m)

Anterior-posterior axis (m)

Anterior-posterior axis (m)

Anterior-posterior axis (m)

Anterior-posterior axis (m)

Anterior-posterior axis (m)

Anterior-posterior axis (m)

Anterior-posterior axis (m)

Anterior-posterior axis (m)

Anterior-posterior axis (m)

Anterior-posterior axis (m)

Anterior-posterior axis (m)

Anterior-posterior axis (m)

Anterior-posterior axis (m)

Anterior-posterior axis (m)

Anterior-posterior axis (m)

Anterior-posterior axis (m)

Anterior-posterior axis (m)

Anterior-posterior axis (m)

Anterior-posterior axis (m)

Anterior-posterior axis (m)

Anterior-posterior axis (m)

Anterior-posterior axis (m)

Anterior-posterior axis (m)

Anterior-posterior axis (m)

Anterior-posterior axis (m)

Anterior-posterior axis (m)

Anterior-posterior axis (m)

Anterior-posterior axis (m)

Anterior-posterior axis (m)

Anterior-posterior axis (m)

Anterior-posterior axis (m)

Anterior-posterior axis (m)

Anterior-posterior axis (m)

Anterior-posterior axis (m)

Anterior-posterior axis (m)

Anterior-posterior axis (m)

Anterior-posterior axis (m)

Anterior-posterior axis (m)

Anterior-posterior axis (m)

Anterior-posterior axis (m)

Anterior-posterior axis (m)

Anterior-posterior axis (m)

Anterior-posterior axis (m)

Anterior-posterior axis (m)

Anterior-posterior axis (m)

Anterior-posterior axis (m)

Anterior-posterior axis (m)

Anterior-posterior axis (m)

Anterior-posterior axis (m)

Anterior-posterior axis (m)

Anterior-posterior axis (m)

Anterior-posterior axis (m)

Anterior-posterior axis (m)

Anterior-posterior axis (m)

Anterior-posterior axis (m)

Anterior-posterior axis (m)

Anterior-posterior axis (m)

Anterior-posterior axis (m)

Anterior-posterior axis (m)

Anterior-posterior axis (m)

Anterior-posterior axis (m)

Anterior-posterior axis (m)

Anterior-posterior axis (m)

Anterior-posterior axis (m)

Anterior-posterior axis (m)

Anterior-posterior axis (m)

Anterior-posterior axis (m)

Anterior-posterior axis (m)

Anterior-posterior axis (m)

Anterior-posterior axis (m)

Anterior-posterior axis (m)

Anterior-posterior axis (m)

Anterior-posterior axis (m)

Anterior-posterior axis (m)

Anterior-posterior axis (m)

Anterior-posterior axis (m)

Anterior-posterior axis (m)

Anterior-posterior axis (m)

Anterior-posterior axis (m)

Anterior-posterior axis (m)

Anterior-posterior axis (m)

Anterior-posterior axis (m)

Anterior-posterior axis (m)

Anterior-posterior axis (m)

Anterior-posterior axis (m)

Anterior-posterior axis (m)

Anterior-posterior axis (m)

Anterior-posterior axis (m)

Anterior-posterior axis (m)

Anterior-posterior axis (m)

Anterior-posterior axis (m)

Anterior-posterior axis (m)

Anterior-posterior axis (m)

Anterior-posterior axis (m)

Anterior-posterior axis (m)

Anterior-posterior axis (m)

Anterior-posterior axis (m)

Anterior-posterior axis (m)

Anterior-posterior axis (m)

Anterior-posterior axis (m)

Anterior-posterior axis (m)

Anterior-posterior axis (m)

Anterior-posterior axis (m)

Anterior-posterior axis (m)

Anterior-posterior axis (m)

Anterior-posterior axis (m)

Anterior-posterior axis (m)

Anterior-posterior axis (m)

Anterior-posterior axis (m)

Anterior-posterior axis (m)

Anterior-posterior axis (m)

Anterior-posterior axis (m)

Anterior-posterior axis (m)

Anterior-posterior axis (m)

Anterior-posterior axis (m)

Anterior-posterior axis (m)

Anterior-posterior axis (m)

Anterior-posterior axis (m)

Anterior-posterior axis (m)

Anterior-posterior axis (m)

Anterior-posterior axis (m)

Anterior-posterior axis (m)

Anterior-posterior axis (m)

Anterior-posterior axis (m)

Anterior-posterior axis (m)

Anterior-posterior axis (m)

Anterior-posterior axis (m)

Anterior-posterior axis (m)

Anterior-posterior axis (m)

Anterior-posterior axis (m)

Anterior-posterior axis (m)

Anterior-posterior axis (m)

Anterior-posterior axis (m)

Anterior-posterior axis (m)

Anterior-posterior axis (m)

Anterior-posterior axis (m)

Anterior-posterior axis (m)

Anterior-posterior axis (m)

Anterior-posterior axis (m)

Anterior-posterior axis (m)

Anterior-posterior axis (m)

Anterior-posterior axis (m)

Anterior-posterior axis (m)

Anterior-posterior axis (m)

Anterior-posterior axis (m)

Anterior-posterior axis (m)

Anterior-posterior axis (m)

Anterior-posterior axis (m)

Anterior-posterior axis (m)

Anterior-posterior axis (m)

Anterior-posterior axis (m)

Anterior-posterior axis (m)

Anterior-posterior axis (m)

Anterior-posterior axis (m)

Anterior-posterior axis (m)

Anterior-posterior axis (m)

Anterior-posterior axis (m)

Anterior-posterior axis (m)

Anterior-posterior axis (m)

Anterior-posterior axis (m)

Anterior-posterior axis (m)

Anterior-posterior axis (m)

Anterior-posterior axis (m)

Anterior-posterior axis (m)

Anterior-posterior axis (m)

Anterior-posterior axis (m)

Anterior-posterior axis (m)

Anterior-posterior axis (m)

Anterior-posterior axis (m)

Anterior-posterior axis (m)

Anterior-posterior axis (m)

Anterior-posterior axis (m)

Anterior-posterior axis (m)

Anterior-posterior axis (m)

Anterior-posterior axis (m)

Anterior-posterior axis (m)

Anterior-posterior axis (m)

Anterior-posterior axis (m)

Anterior-posterior axis (m)

Anterior-posterior axis (m)

Anterior-posterior axis (m)

Anterior-posterior axis (m)

Anterior-posterior axis (m)

Anterior-posterior axis (m)

Anterior-posterior axis (m)

Anterior-posterior axis (m)

Anterior-posterior axis (m)

Anterior-posterior axis (m)

Anterior-posterior axis (m)

Anterior-posterior axis (m)

Anterior-posterior axis (m)

Anterior-posterior axis (m)

Anterior-posterior axis (m)

Anterior-posterior axis (m)

Anterior-posterior axis (m)

Anterior-posterior axis (m)

Anterior-posterior axis (m)

Anterior-posterior axis (m)

Anterior-posterior axis (m)

Anterior-posterior axis (m)

Anterior-posterior axis (m)

Anterior-posterior axis (m)

Anterior-posterior axis (m)

Anterior-posterior axis (m)

Anterior-posterior axis (m)

Anterior-posterior axis (m)

Anterior-posterior axis (m)

Anterior-posterior axis (m)

Anterior-posterior axis (m)

Anterior-posterior axis (m)

Anterior-posterior axis (m)

Anterior-posterior axis (m)

Anterior-posterior axis (m)

Anterior-posterior axis (m)

Anterior-posterior axis (m)

Anterior-posterior axis (m)

Anterior-posterior axis (m)

Anterior-posterior axis (m)

Anterior-posterior axis (m)

Anterior-posterior axis (m)

Anterior-posterior axis (m)

Anterior-posterior axis (m)

Anterior-posterior axis (m)

Anterior-posterior axis (m)

Anterior-posterior axis (m)

Anterior-posterior axis (m)

Anterior-posterior axis (m)

Anterior-posterior axis (m)

Anterior-posterior axis (m)

Anterior-posterior axis (m)

Anterior-posterior axis (m)

Anterior-posterior axis (m)

Anterior-posterior axis (m)

Anterior-posterior axis (m)

Anterior-posterior axis (m)

Anterior-posterior axis (m)

Anterior-posterior axis (m)

Anterior-posterior axis (m)

Anterior-posterior axis (m)

Anterior-posterior axis (m)

Anterior-posterior axis (m)

Anterior-posterior axis (m)

Anterior-posterior axis (m)

Anterior-posterior axis (m)

Anterior-posterior axis (m)

Anterior-posterior axis (m)

Anterior-posterior axis (m)

Anterior-posterior axis (m)

Anterior-posterior axis (m)

Anterior-posterior axis (m)

Anterior-posterior axis (m)

Anterior-posterior axis (m)

Anterior-posterior axis (m)

Anterior-posterior axis (m)

Anterior-posterior axis (m)

Anterior-posterior axis (m)

Anterior-posterior axis (m)

Anterior-posterior axis (m)

Anterior-posterior axis (m)

Anterior-posterior axis (m)

Anterior-posterior axis (m)

Anterior-posterior axis (m)

Anterior-posterior axis (m)

Anterior-posterior axis (m)

Anterior-posterior axis (m)

Anterior-posterior axis (m)

Anterior-posterior axis (m)

Anterior-posterior axis (m)

Anterior-posterior axis (m)

Anterior-posterior axis (m)

Anterior-posterior axis (m)

Anterior-posterior axis (m)

Anterior-posterior axis (m)

Anterior-posterior axis (m)

Anterior-posterior axis (m)

Anterior-posterior axis (m)

Anterior-posterior axis (m)

Anterior-posterior axis (m)

Anterior-posterior axis (m)

Anterior-posterior axis (m)

Anterior-posterior axis (m)

Anterior-posterior axis (m)

Anterior-posterior axis (m)

Anterior-posterior axis (m)

Anterior-posterior axis (m)

Anterior-posterior axis (m)

Anterior-posterior axis (m)

Anterior-posterior axis (m)

Anterior-posterior axis (m)

Anterior-posterior axis (m)

Anterior-posterior axis (m)

Anterior-posterior axis (m)

Anterior-posterior axis (m)

Anterior-posterior axis (m)

Anterior-posterior axis (m)

Anterior-posterior axis (m)

Anterior-posterior axis (m)

Anterior-posterior axis (m)

Anterior-posterior axis (m)

Anterior-posterior axis (m)

Anterior-posterior axis (m)

Anterior-posterior axis (m)

Anterior-posterior axis (m)

Anterior-posterior axis (m)

Anterior-posterior axis (m)

Anterior-posterior axis (m)

Anterior-posterior axis (m)

Anterior-posterior axis (m)

Anterior-posterior axis (m)

Anterior-posterior axis (m)

Anterior-posterior axis (m)

Anterior-posterior axis (m)

Anterior-posterior axis (m)

Anterior-posterior axis (m)

Anterior-posterior axis (m)

Anterior-posterior axis (m)

Anterior-posterior axis (m)

Anterior-posterior axis (m)

Anterior-posterior axis (m)

Anterior-posterior axis (m)

Anterior-posterior axis (m)

Anterior-posterior axis (m)

Anterior-posterior axis (m)

Anterior-posterior axis (m)

Anterior-posterior axis (m)

Anterior-posterior axis (m)

Anterior-posterior axis (m)

Anterior-posterior axis (m)

Anterior-posterior axis (m)

Anterior-posterior axis (m)

Anterior-posterior axis (m)

Anterior-posterior axis (m)

Anterior-posterior axis (m)

Anterior-posterior axis (m)

Anterior-posterior axis (m)

Anterior-posterior axis (m)

Anterior-posterior axis (m)

Anterior-posterior axis (m)

Anterior-posterior axis (m)

Anterior-posterior axis (m)

Anterior-posterior axis (m)

Anterior-posterior axis (m)

Anterior-posterior axis (m)

Anterior-posterior axis (m)

Anterior-posterior axis (m)

Anterior-posterior axis (m)

Anterior-posterior axis (m)

Anterior-posterior axis (m)

Anterior-posterior axis (m)

Anterior-posterior axis (m)

Anterior-posterior axis (m)

Anterior-posterior axis (m)

Anterior-posterior axis (m)

Anterior-posterior axis (m)

Anterior-posterior axis (m)

Anterior-posterior axis (m)

Anterior-posterior axis (m)

Anterior-posterior axis (m)

Anterior-posterior axis (m)

Anterior-posterior axis (m)

Anterior-posterior axis (m)

Anterior-posterior axis (m)

Anterior-posterior axis (m)

Anterior-posterior axis (m)

Anterior-posterior axis (m)

Anterior-posterior axis (m)

Anterior-posterior axis (m)

Anterior-posterior axis (m)

Anterior-posterior axis (m)

Anterior-posterior axis (m)

Anterior-posterior axis (m)

Anterior-posterior axis (m)

Anterior-posterior axis (m)

Anterior-posterior axis (m)

Anterior-posterior axis (m)

Anterior-posterior axis (m)

Anterior-posterior axis (m)

Anterior-posterior axis (m)

Anterior-posterior axis (m)

Anterior-posterior axis (m)

Anterior-posterior axis (m)

Anterior-posterior axis (m)

Anterior-posterior axis (m)

Anterior-posterior axis (m)

Anterior-posterior axis (m)

Anterior-posterior axis (m)

Anterior-posterior axis (m)

Anterior-posterior axis (m)

Anterior-posterior axis (m)

Anterior-posterior axis (m)

Anterior-posterior axis (m)

Anterior-posterior axis (m)

Anterior-posterior axis (m)

Anterior-posterior axis (m)

Anterior-posterior axis (m)

Anterior-posterior axis (m)

Anterior-posterior axis (m)

Anterior-posterior axis (m)

Anterior-posterior axis (m)

Anterior-posterior axis (m)

Anterior-posterior axis (m)

Anterior-posterior axis (m)

Anterior-posterior axis (m)

Anterior-posterior axis (m)

Anterior-posterior axis (m)

Anterior-posterior axis (m)

Anterior-posterior axis (m)

Anterior-posterior axis (m)

Anterior-posterior axis (m)

Anterior-posterior axis (m)

Anterior-posterior axis (m)

Anterior-posterior axis (m)

Anterior-posterior axis (m)

Anterior-posterior axis (m)

Anterior-posterior axis (m)

Anterior-posterior axis (m)

Anterior-posterior axis (m)

Anterior-posterior axis (m)

Anterior-posterior axis (m)

Anterior-posterior axis (m)

Anterior-posterior axis (m)

Anterior-posterior axis (m)

Anterior-posterior axis (m)

Anterior-posterior axis (m)

Anterior-posterior axis (m)

Anterior-posterior axis (m)

Anterior-posterior axis (m)

Anterior-posterior axis (m)

Anterior-posterior axis (m)

Anterior-posterior axis (m)

Anterior-posterior axis (m)

Anterior-posterior axis (m)

Anterior-posterior axis (m)

Anterior-posterior axis (m)

Anterior-posterior axis (m)

Anterior-posterior axis (m)

Anterior-posterior axis (m)

Anterior-posterior axis (m)

Anterior-posterior axis (m)

Anterior-posterior axis (m)

Anterior-posterior axis (m)

Anterior-posterior axis (m)

Anterior-posterior axis (m)

Anterior-posterior axis (m)

Anterior-posterior axis (m)

Anterior-posterior axis (m)

Anterior-posterior axis (m)

Anterior-posterior axis (m)

Anterior-posterior axis (m)

Anterior-posterior axis (m)

Anterior-posterior axis (m)

Anterior-posterior axis (m)

Anterior-posterior axis (m)

Anterior-posterior axis (m)

Anterior-posterior axis (m)

Anterior-posterior axis (m)

Anterior-posterior axis (m)

Anterior-posterior axis (m)

Anterior-posterior axis (m)

Anterior-posterior axis (m)

Anterior-posterior axis (m)

Anterior-posterior axis (m)

Anterior-posterior axis (m)

Anterior-posterior axis (m)

Anterior-posterior axis (m)

Anterior-posterior axis (m)

Anterior-posterior axis (m)

Anterior-posterior axis (m)

Anterior-posterior axis (m)

Anterior-posterior axis (m)

Anterior-posterior axis (m)

Anterior-posterior axis (m)

Anterior-posterior axis (m)

Anterior-posterior axis (m)

Anterior-posterior axis (m)

Anterior-posterior axis (m)

Anterior-posterior axis (m)

Anterior-posterior axis (m)

Anterior-posterior axis (m)

Anterior-posterior axis (m)

Anterior-posterior axis (m)

Anterior-posterior axis (m)

Anterior-posterior axis (m)

Anterior-posterior axis (m)

Anterior-posterior axis (m)

Anterior-posterior axis (m)

Anterior-posterior axis (m)

Anterior-posterior axis (m)

Anterior-posterior axis (m)

Anterior-posterior axis (m)

Anterior-posterior axis (m)

Anterior-posterior axis (m)

Anterior-posterior axis (m)

Anterior-posterior axis (m)

Anterior-posterior axis (m)

Anterior-posterior axis (m)

Anterior-posterior axis (m)

Anterior-posterior axis (m)

Anterior-posterior axis (m)

Anterior-posterior axis (m)

Anterior-posterior axis (m)

Anterior-posterior axis (m)

Anterior-posterior axis (m)

Anterior-posterior axis (m)

Anterior-posterior axis (m)

Anterior-posterior axis (m)

Anterior-posterior axis (m)

Anterior-posterior axis (m)

Anterior-posterior axis (m)

Anterior-posterior axis (m)

Anterior-posterior axis (m)

Anterior-posterior axis (m)

Anterior-posterior axis (m)

Anterior-posterior axis (m)

Anterior-posterior axis (m)

Anterior-posterior axis (m)

Anterior-posterior axis (m)

Anterior-posterior axis (m)

Anterior-posterior axis (m)

Anterior-posterior axis (m)

Anterior-posterior axis (m)

Anterior-posterior axis (m)

Anterior-posterior axis (m)

Anterior-posterior axis (m)

Anterior-posterior axis (m)

Anterior-posterior axis (m)

Anterior-posterior axis (m)

Anterior-posterior axis (m)

Anterior-posterior axis (m)

Anterior-posterior axis (m)

Anterior-posterior axis (m)

Anterior-posterior axis (m)

Anterior-posterior axis (m)

Anterior-posterior axis (m)

Anterior-posterior axis (m)

Anterior-posterior axis (m)

Anterior-posterior axis (m)

Anterior-posterior axis (m)

Anterior-posterior axis (m)

Anterior-posterior axis (m)

Anterior-posterior axis (m)

Anterior-posterior axis (m)

Anterior-posterior axis (m)

Anterior-posterior axis (m)

Anterior-posterior axis (m)

Anterior-posterior axis (m)

Anterior-posterior axis (m)

Anterior-posterior axis (m)

Anterior-posterior axis (m)

Anterior-posterior axis (m)

Anterior-posterior axis (m)

Anterior-posterior axis (m)

Anterior-posterior axis (m)

Anterior-posterior axis (m)

Anterior-posterior axis (m)

Anterior-posterior axis (m)

Anterior-posterior axis (m)

Anterior-posterior axis (m)

Anterior-posterior axis (m)

Anterior-posterior axis (m)

Anterior-posterior axis (m)

Anterior-posterior axis (m)

Anterior-posterior axis (m)

Anterior-posterior axis (m)

Anterior-posterior axis (m)

Anterior-posterior axis (m)

Anterior-posterior axis (m)

Anterior-posterior axis (m)

Anterior-posterior axis (m)

Anterior-posterior axis (m)

Anterior-posterior axis (m)

Anterior-posterior axis (m)

Anterior-posterior axis (m)

Anterior-posterior axis (m)

Anterior-posterior axis (m)

Anterior-posterior axis (m)

Anterior-posterior axis (m)

Anterior-posterior axis (m)

Anterior-posterior axis (m)

Anterior-posterior axis (m)

Anterior-posterior axis (m)

Anterior-posterior axis (m)

Anterior-posterior axis (m)

Anterior-posterior axis (m)

Anterior-posterior axis (m)

Anterior-posterior axis (m)

Anterior-posterior axis (m)

Anterior-posterior axis (m)

Anterior-posterior axis (m)

Anterior-posterior axis (m)

Anterior-posterior axis (m)

Anterior-posterior axis (m)

Anterior-posterior axis (m)

Anterior-posterior axis (m)

Anterior-posterior axis (m)

Anterior-posterior axis (m)

Anterior-posterior axis (m)

Anterior-posterior axis (m)

Anterior-posterior axis (m)

Anterior-posterior axis (m)

Anterior-posterior axis (m)

Anterior-posterior axis (m)

Anterior-posterior axis (m)

Anterior-posterior axis (m)

Anterior-posterior axis (m)

Anterior-posterior axis (m)

Anterior-posterior axis (m)

Anterior-posterior axis (m)

Anterior-posterior axis (m)

Anterior-posterior axis (m)

Anterior-posterior axis (m)

Anterior-posterior axis (m)

Anterior-posterior axis (m)

Anterior-posterior axis (m)

Anterior-posterior axis (m)

Anterior-posterior axis (m)

Anterior-posterior axis (m)

Anterior-posterior axis (m)

Anterior-posterior axis (m)

Anterior-posterior axis (m)

Anterior-posterior axis (m)

Anterior-posterior axis (m)

Anterior-posterior axis (m)

Anterior-posterior axis (m)

Anterior-posterior axis (m)

Anterior-posterior axis (m)

Anterior-posterior axis (m)

Anterior-posterior axis (m)

Anterior-posterior axis (m)

Anterior-posterior axis (m)

Anterior-posterior axis (m)

Anterior-posterior axis (m)

Anterior-posterior axis (m)

Anterior-posterior axis (m)

Anterior-posterior axis (m)

Anterior-posterior axis (m)

Anterior-posterior axis (m)

Anterior-posterior axis (m)

Anterior-posterior axis (m)

Anterior-posterior axis (m)

Anterior-posterior axis (m)

Anterior-posterior axis (m)

Anterior-posterior axis (m)

Anterior-posterior axis (m)

Anterior-posterior axis (m)

Anterior-posterior axis (m)

Anterior-posterior axis (m)

Anterior-posterior axis (m)

Anterior-posterior axis (m)

Anterior-posterior axis (m)

Anterior-posterior axis (m)

Anterior-posterior axis (m)

Anterior-posterior axis (m)

Anterior-posterior axis (m)

Anterior-posterior axis (m)

Anterior-posterior axis (m)

Anterior-posterior axis (m)

Anterior-posterior axis (m)

Anterior-posterior axis (m)

Anterior-posterior axis (m)

Anterior-posterior axis (m)

Anterior-posterior axis (m)

Anterior-posterior axis (m)

Anterior-posterior axis (m)

Anterior-posterior axis (m)

Anterior-posterior axis (m)

Anterior-posterior axis (m)

Anterior-posterior axis (m)

Anterior-posterior axis (m)

Anterior-posterior axis (m)

Anterior-posterior axis (m)

Anterior-posterior axis (m)

Anterior-posterior axis (m)

Anterior-posterior axis (m)

Anterior-posterior axis (m)

Anterior-posterior axis (m)

Anterior-posterior axis (m)

Anterior-posterior axis (m)

Anterior-posterior axis (m)

Anterior-posterior axis (m)

Anterior-posterior axis (m)

Anterior-posterior axis (m)

Anterior-posterior axis (m)

Anterior-posterior axis (m)

Anterior-posterior axis (m)

Anterior-posterior axis (m)

Anterior-posterior axis (m)

Anterior-posterior axis (m)

Anterior-posterior axis (m)

Anterior-posterior axis (m)

Anterior-posterior axis (m)

Anterior-posterior axis (m)

Anterior-posterior axis (m)

Anterior-posterior axis (m)

Anterior-posterior axis (m)

Anterior-posterior axis (m)

Anterior-posterior axis (m)

Anterior-posterior axis (m)

Anterior-posterior axis (m)

Anterior-posterior axis (m)

Anterior-posterior axis (m)

Anterior-posterior axis (m)

Anterior-posterior axis (m)

Anterior-posterior axis (m)

Anterior-posterior axis (m)

Anterior-posterior axis (m)

Anterior-posterior axis (m)

Anterior-posterior axis (m)

Anterior-posterior axis (m)

Anterior-posterior axis (m)

Anterior-posterior axis (m)

Anterior-posterior axis (m)

Anterior-posterior axis (m)

Anterior-posterior axis (m)

Anterior-posterior axis (m)

Anterior-posterior axis (m)

Anterior-posterior axis (m)

Anterior-posterior axis (m)

Anterior-posterior axis (m)

Anterior-posterior axis (m)

Anterior-posterior axis (m)

Anterior-posterior axis (m)

Anterior-posterior axis (m)

Anterior-posterior axis (m)

Anterior-posterior axis (m)

Anterior-posterior axis (m)

Anterior-posterior axis (m)

Anterior-posterior axis (m)

Anterior-posterior axis (m)

Anterior-posterior axis (m)

Anterior-posterior axis (m)

Anterior-posterior axis (m)

Anterior-posterior axis (m)

Anterior-posterior axis (m)

Anterior-posterior axis (m)

Anterior-posterior axis (m)

Anterior-posterior axis (m)

Anterior-posterior axis (m)

Anterior-posterior axis (m)

Anterior-posterior axis (m)

Anterior-posterior axis (m)

Anterior-posterior axis (m)

Anterior-posterior axis (m)

Anterior-posterior axis (m)

Anterior-posterior axis (m)

Anterior-posterior axis (m)

Anterior-posterior axis (m)

Anterior-posterior axis (m)

Anterior-posterior axis (m)

Anterior-posterior axis (m)

Anterior-posterior axis (m)

Anterior-posterior axis (m)

Anterior-posterior axis (m)

Anterior-posterior axis (m)

Anterior-posterior axis (m)

Anterior-posterior axis (m)

Anterior-posterior axis (m)

Anterior-posterior axis (m)

Anterior-posterior axis (m)

Anterior-posterior axis (m)

Anterior-posterior axis (m)

Anterior-posterior axis (m)

Anterior-posterior axis (m)

Anterior-posterior axis (m)

Anterior-posterior axis (m)

Anterior-posterior axis (m)

Anterior-posterior axis (m)

Anterior-posterior axis (m)

Anterior-posterior axis (m)

Anterior-posterior axis (m)

Anterior-posterior axis (m)

Anterior-posterior axis (m)

Anterior-posterior axis (m)

Anterior-posterior axis (m)

Anterior-posterior axis (m)

Anterior-posterior axis (m)

Anterior-posterior axis (m)

Anterior-posterior axis (m)

Anterior-posterior axis (m)

Anterior-posterior axis (m)

Anterior-posterior axis (m)

Anterior-posterior axis (m)

Anterior-posterior axis (m)

Anterior-posterior axis (m)

Anterior-posterior axis (m)

Anterior-posterior axis (m)

Anterior-posterior axis (m)

Anterior-posterior axis (m)

Anterior-posterior axis (m)

Anterior-posterior axis (m)

Anterior-posterior axis (m)

Anterior-posterior axis (m)

Anterior-posterior axis (m)

Anterior-posterior axis (m)

Anterior-posterior axis (m)

Anterior-posterior axis (m)

Anterior-posterior axis (m)

Anterior-posterior axis (m)

Anterior-posterior axis (m)

Anterior-posterior axis (m)

Anterior-posterior axis (m)

Anterior-posterior axis (m)

Anterior-posterior axis (m)

Anterior-posterior axis (m)

Anterior-posterior axis (m)

Anterior-posterior axis (m)

Anterior-posterior axis (m)

Anterior-posterior axis (m)

Anterior-posterior axis (m)

Anterior-posterior axis (m)

Anterior-posterior axis (m)

Anterior-posterior axis (m)

Anterior-posterior axis (m)

Anterior-posterior axis (m)

Anterior-posterior axis (m)

Anterior-posterior axis (m)

Anterior-posterior axis (m)

Anterior-posterior axis (m)

Anterior-posterior axis (m)

Anterior-posterior axis (m)

Anterior-posterior axis (m)

Anterior-posterior axis (m)

Anterior-posterior axis (m)

Anterior-posterior axis (m)

Anterior-posterior axis (m)

Anterior-posterior axis (m)

Anterior-posterior axis (m)

Anterior-posterior axis (m)

Anterior-posterior axis (m)

Anterior-posterior axis (m)

Anterior-posterior axis (m)

Anterior-posterior axis (m)

Anterior-posterior axis (m)

Anterior-posterior axis (m)

Anterior-posterior axis (m)

Anterior-posterior axis (m)

Anterior-posterior axis (m)

Anterior-posterior axis (m)

Anterior-posterior axis (m)

Anterior-posterior axis (m)

Anterior-posterior axis (m)

Anterior-posterior axis (m)

Anterior-posterior axis (m)

Anterior-posterior axis (m)

Anterior-posterior axis (m)

Anterior-posterior axis (m)

Anterior-posterior axis (m)

Anterior-posterior axis (m)

Anterior-posterior axis (m)

Anterior-posterior axis (m)

Anterior-posterior axis (m)

Anterior-posterior axis (m)

Anterior-posterior axis (m)

Anterior-posterior axis (m)

Anterior-posterior axis (m)

Anterior-posterior axis (m)

Anterior-posterior axis (m)

Anterior-posterior axis (m)

Anterior-posterior axis (m)

Anterior-posterior axis (m)

Anterior-posterior axis (m)

Anterior-posterior axis (m)

Anterior-posterior axis (m)

Anterior-posterior axis (m)

Anterior-posterior axis (m)

Anterior-posterior axis (m)

Anterior-posterior axis (m)

Anterior-posterior axis (m)

Anterior-posterior axis (m)

Anterior-posterior axis (m)

Anterior-posterior axis (m)

Anterior-posterior axis (m)

Anterior-posterior axis (m)

Anterior-posterior axis (m)

Anterior-posterior axis (m)

Anterior-posterior axis (m)

Anterior-posterior axis (m)

Anterior-posterior axis (m)

Anterior-posterior axis (m)

Anterior-posterior axis (m)

Anterior-posterior axis (m)

Anterior-posterior axis (m)

Anterior-posterior axis (m)

Anterior-posterior axis (m)

Anterior-posterior axis (m)

Anterior-posterior axis (m)

Anterior-posterior axis (m)

Anterior-posterior axis (m)

Anterior-posterior axis (m)

Anterior-posterior axis (m)

Anterior-posterior axis (m)

Anterior-posterior axis (m)

Anterior-posterior axis (m)

Anterior-posterior axis (m)

Anterior-posterior axis (m)

Anterior-posterior axis (m)

Anterior-posterior axis (m)

Anterior-posterior axis (m)

Anterior-posterior axis (m)

Anterior-posterior axis (m)

Anterior-posterior axis (m)

Anterior-posterior axis (m)

Anterior-posterior axis (m)

Anterior-posterior axis (m)

Anterior-posterior axis (m)

Anterior-posterior axis (m)

Anterior-posterior axis (m)

Anterior-posterior axis (m)

Anterior-posterior axis (m)

Anterior-posterior axis (m)

Anterior-posterior axis (m)

Anterior-posterior axis (m)

Anterior-posterior axis (m)

Anterior-posterior axis (m)

Anterior-posterior axis (m)

Anterior-posterior axis (m)

Anterior-posterior axis (m)

Anterior-posterior axis (m)

Anterior-posterior axis (m)

Anterior-posterior axis (m)

Anterior-posterior axis (m)

Anterior-posterior axis (m)

Anterior-posterior axis (m)

Anterior-posterior axis (m)

Anterior-posterior axis (m)

Anterior-posterior axis (m)

Anterior-posterior axis (m)

Anterior-posterior axis (m)

Anterior-posterior axis (m)

Anterior-posterior axis (m)

Anterior-posterior axis (m)

Anterior-posterior axis (m)

Anterior-posterior axis (m)

Anterior-posterior axis (m)

Anterior-posterior axis (m)

Anterior-posterior axis (m)

Anterior-posterior axis (m)

Anterior-posterior axis (m)

Anterior-posterior axis (m)

Anterior-posterior axis (m)

Anterior-posterior axis (m)

Anterior-posterior axis (m)

Anterior-posterior axis (m)

Anterior-posterior axis (m)

Anterior-posterior axis (m)

Anterior-posterior axis (m)

Anterior-posterior axis (m)

Anterior-posterior axis (m)

Anterior-posterior axis (m)

Anterior-posterior axis (m)

Anterior-posterior axis (m)

Anterior-posterior axis (m)

Anterior-posterior axis (m)

Anterior-posterior axis (m)

Anterior-posterior axis (m)

Anterior-posterior axis (m)

Anterior-posterior axis (m)

Anterior-posterior axis (m)

Anterior-posterior axis (m)

Anterior-posterior axis (m)

Anterior-posterior axis (m)

Anterior-posterior axis (m)

Anterior-posterior axis (m)

Anterior-posterior axis (m)

Anterior-posterior axis (m)

Anterior-posterior axis (m)

Anterior-posterior axis (m)

Anterior-posterior axis (m)

Anterior-posterior axis (m)

Anterior-posterior axis (m)

Anterior-posterior axis (m)

Anterior-posterior axis (m)

Anterior-posterior axis (m)

Anterior-posterior axis (m)

Anterior-posterior axis (m)

Anterior-posterior axis (m)

Anterior-posterior axis (m)

Anterior-posterior axis (m)

Anterior-posterior axis (m)

Anterior-posterior axis (m)

Anterior-posterior axis (m)

Anterior-posterior axis (m)

Anterior-posterior axis (m)

Anterior-posterior axis (m)

Anterior-posterior axis (m)

Anterior-posterior axis (m)

Anterior-posterior axis (m)

Anterior-posterior axis (m)

Anterior-posterior axis (m)

Anterior-posterior axis (m)

Anterior-posterior axis (m)

Anterior-posterior axis (m)

Anterior-posterior axis (m)

Anterior-posterior axis (m)

Anterior-posterior axis (m)

Anterior-posterior axis (m)

Anterior-posterior axis (m)

Anterior-posterior axis (m)

Anterior-posterior axis (m)

Anterior-posterior axis (m)

Anterior-posterior axis (m)

Anterior-posterior axis (m)

Anterior-posterior axis (m)

Anterior-posterior axis (m)

Anterior-posterior axis (m)

Anterior-posterior axis (m)

Anterior-posterior axis (m)

Anterior-posterior axis (m)

Anterior-posterior axis (m)

Anterior-posterior axis (m)

Anterior-posterior axis (m)

Anterior-posterior axis (m)

Anterior-posterior axis (m)

Anterior-posterior axis (m)

Anterior-posterior axis (m)

Anterior-posterior axis (m)

Anterior-posterior axis (m)

Anterior-posterior axis (m)

Anterior-posterior axis (m)

Anterior-posterior axis (m)

Anterior-posterior axis (m)

Anterior-posterior axis (m)

Anterior-posterior axis (m)

Anterior-posterior axis (m)

Anterior-posterior axis (m)

Anterior-posterior axis (m)

Anterior-posterior axis (m)

Anterior-posterior axis (m)

Anterior-posterior axis (m)

Anterior-posterior axis (m)

Anterior-posterior axis (m)

Anterior-posterior axis (m)

Anterior-posterior axis (m)

Anterior-posterior axis (m)

Anterior-posterior axis (m)

Anterior-posterior axis (m)

Anterior-posterior axis (m)

Anterior-posterior axis (m)

Anterior-posterior axis (m)

Anterior-posterior axis (m)

Anterior-posterior axis (m)

Anterior-posterior axis (m)

Anterior-posterior axis (m)

Anterior-posterior axis (m)

Anterior-posterior axis (m)

Anterior-posterior axis (m)

Anterior-posterior axis (m)

Anterior-posterior axis (m)

Anterior-posterior axis (m)

Anterior-posterior axis (m)

Anterior-posterior axis (m)

Anterior-posterior axis (m)

Anterior-posterior axis (m)

Anterior-posterior axis (m)

Anterior-posterior axis (m)

Anterior-posterior axis (m)

Anterior-posterior axis (m)

Anterior-posterior axis (m)

Anterior-posterior axis (m)

Anterior-posterior axis (m)

Anterior-posterior axis (m)

Anterior-posterior axis (m)

Anterior-posterior axis (m)

Anterior-posterior axis (m)

Anterior-posterior axis (m)

Anterior-posterior axis (m)

Anterior-posterior axis (m)

Anterior-posterior axis (m)

Anterior-posterior axis (m)

Anterior-posterior axis (m)

Anterior-posterior axis (m)

Anterior-posterior axis (m)

Anterior-posterior axis (m)

Anterior-posterior axis (m)

Anterior-posterior axis (m)

Anterior-posterior axis (m)

Anterior-posterior axis (m)

Anterior-posterior axis (m)

Anterior-posterior axis (m)

Anterior-posterior axis (m)

Anterior-posterior axis (m)

Anterior-posterior axis (m)

Anterior-posterior axis (m)

Anterior-posterior axis (m)

Anterior-posterior axis (m)

Anterior-posterior axis (m)

Anterior-posterior axis (m)

Anterior-posterior axis (m)

Anterior-posterior axis (m)

Anterior-posterior axis (m)

Anterior-posterior axis (m)

Anterior-posterior axis (m)

Anterior-posterior axis (m)

Anterior-posterior axis (m)

Anterior-posterior axis (m)

Anterior-posterior axis (m)

Anterior-posterior axis (m)

Anterior-posterior axis (m)

Anterior-posterior axis (m)

Anterior-posterior axis (m)

Anterior-posterior axis (m)

Anterior-posterior axis (m)

Anterior-posterior axis (m)

Anterior-posterior axis (m)

Anterior-posterior axis (m)

Anterior-posterior axis (m)

Anterior-posterior axis (m)

Anterior-posterior axis (m)

Anterior-posterior axis (m)

Anterior-posterior axis (m)

Anterior-posterior axis (m)

Anterior-posterior axis (m)

Anterior-posterior axis (m)

Anterior-posterior axis (m)

Anterior-posterior axis (m)

Anterior-posterior axis (m)

Anterior-posterior axis (m)

Anterior-posterior axis (m)

Anterior-posterior axis (m)

Anterior-posterior axis (m)

Anterior-posterior axis (m)

Anterior-posterior axis (m)

Anterior-posterior axis (m)

Anterior-posterior axis (m)

Anterior-posterior axis (m)

Anterior-posterior axis (m)

Anterior-posterior axis (m)

Anterior-posterior axis (m)

Anterior-posterior axis (m)

Anterior-posterior axis (m)

Anterior-posterior axis (m)

Anterior-posterior axis (m)

Anterior-posterior axis (m)

Anterior-posterior axis (m)

Anterior-posterior axis (m)

Anterior-posterior axis (m)

Anterior-posterior axis (m)

Anterior-posterior axis (m)

Anterior-posterior axis (m)

Anterior-posterior axis (m)

Anterior-posterior axis (m)

Anterior-posterior axis (m)

Anterior-posterior axis (m)

Anterior-posterior axis (m)

Anterior-posterior axis (m)

Anterior-posterior axis (m)

Anterior-posterior axis (m)

Anterior-posterior axis (m)

Anterior-posterior axis (m)

Anterior-posterior axis (m)

Anterior-posterior axis (m)

Anterior-posterior axis (m)

Anterior-posterior axis (m)

Anterior-posterior axis (m)

Anterior-posterior axis (m)

Anterior-posterior axis (m)

Anterior-posterior axis (m)

Anterior-posterior axis (m)

Anterior-posterior axis (m)

Anterior-posterior axis (m)

Anterior-posterior axis (m)

Anterior-posterior axis (m)

Anterior-posterior axis (m)

Anterior-posterior axis (m)

Anterior-posterior axis (m)

Anterior-posterior axis (m)

Anterior-posterior axis (m)

Anterior-posterior axis (m)

Anterior-posterior axis (m)

Anterior-posterior axis (m)

Anterior-posterior axis (m)

Anterior-posterior axis (m)

Anterior-posterior axis (m)

Anterior-posterior axis (m)

Anterior-posterior axis (m)

Anterior-posterior axis (m)

Anterior-posterior axis (m)

Anterior-posterior axis (m)

Anterior-posterior axis (m)

Anterior-posterior axis (m)

Anterior-posterior axis (m)

Anterior-posterior axis (m)

Anterior-posterior axis (m)

Anterior-posterior axis (m)

Anterior-posterior axis (m)

Anterior-posterior axis (m)

Anterior-posterior axis (m)

Anterior-posterior axis (m)

Anterior-posterior axis (m)

Anterior-posterior axis (m)

Anterior-posterior axis (m)

Anterior-posterior axis (m)

Anterior-posterior axis (m)

Anterior-posterior axis (m)

Anterior-posterior axis (m)

Anterior-posterior axis (m)

Anterior-posterior axis (m)

Anterior-posterior axis (m)

Anterior-posterior axis (m)

Anterior-posterior axis (m)

Anterior-posterior axis (m)

Anterior-posterior axis (m)

Anterior-posterior axis (m)

Anterior-posterior axis (m)

Anterior-posterior axis (m)

Anterior-posterior axis (m)

Anterior-posterior axis (m)

Anterior-posterior axis (m)

Anterior-posterior axis (m)

Anterior-posterior axis (m)

Anterior-posterior axis (m)

Anterior-posterior axis (m)

Anterior-posterior axis (m)

Anterior-posterior axis (m)

Anterior-posterior axis (m)

Anterior-posterior axis (m)

Anterior-posterior axis (m)

Anterior-posterior axis (m)

Anterior-posterior axis (m)

Anterior-posterior axis (m)

Anterior-posterior axis (m)

Anterior-posterior axis (m)

Anterior-posterior axis (m)

Anterior-posterior axis (m)

Anterior-posterior axis (m)

Anterior-posterior axis (m)

Anterior-posterior axis (m)

Anterior-posterior axis (m)

Anterior-posterior axis (m)

Anterior-posterior axis (m)

Anterior-posterior axis (m)

Anterior-posterior axis (m)

Anterior-posterior axis (m)

Anterior-posterior axis (m)

Anterior-posterior axis (m)

Anterior-posterior axis (m)

Anterior-posterior axis (m)

Anterior-posterior axis (m)

Anterior-posterior axis (m)

Anterior-posterior axis (m)

Anterior-posterior axis (m)

Anterior-posterior axis (m)

Anterior-posterior axis (m)

Anterior-posterior axis (m)

Anterior-posterior axis (m)

Anterior-posterior axis (m)

Anterior-posterior axis (m)

Anterior-posterior axis (m)

Anterior-posterior axis (m)

Anterior-posterior axis (m)

Anterior-posterior axis (m)

Anterior-posterior axis (m)

Anterior-posterior axis (m)

Anterior-posterior axis (m)

Anterior-posterior axis (m)

Anterior-posterior axis (m)

Anterior-posterior axis (m)

Anterior-posterior axis (m)

Anterior-posterior axis (m)

Anterior-posterior axis (m)

Anterior-posterior axis (m)

Anterior-posterior axis (m)

Anterior-posterior axis (m)

Anterior-posterior axis (m)

Anterior-posterior axis (m)

Anterior-posterior axis (m)

Anterior-posterior axis (m)

Anterior-posterior axis (m)

Anterior-posterior axis (m)

Anterior-posterior axis (m)

Anterior-posterior axis (m)

Anterior-posterior axis (m)

Anterior-posterior axis (m)

Anterior-posterior axis (m)

Anterior-posterior axis (m)

Anterior-posterior axis (m)

Anterior-posterior axis (m)

Anterior-posterior axis (m)

Anterior-posterior axis (m)

Anterior-posterior axis (m)

Anterior-posterior axis (m)

Anterior-posterior axis (m)

Anterior-posterior axis (m)

Anterior-posterior axis (m)

Anterior-posterior axis (m)

Anterior-posterior axis (m)

Anterior-posterior axis (m)

Anterior-posterior axis (m)

Anterior-posterior axis (m)

Anterior-posterior axis (m)

Anterior-posterior axis (m)

Anterior-posterior axis (m)

Anterior-posterior axis (m)

Anterior-posterior axis (m)

Anterior-posterior axis (m)

Anterior-posterior axis (m)

Anterior-posterior axis (m)

Anterior-posterior axis (m)

Anterior-posterior axis (m)

Anterior-posterior axis (m)

Anterior-posterior axis (m)

Anterior-posterior axis (m)

Anterior-posterior axis (m)

Anterior-posterior axis (m)

Anterior-posterior axis (m)

Anterior-posterior axis (m)

Anterior-posterior axis (m)

Anterior-posterior axis (m)

Anterior-posterior axis (m)

Anterior-posterior axis (m)

Anterior-posterior axis (m)

Anterior-posterior axis (m)

Anterior-posterior axis (m)

Anterior-posterior axis (m)

Anterior-posterior axis (m)

Anterior-posterior axis (m)

Anterior-posterior axis (m)

Anterior-posterior axis (m)

Anterior-posterior axis (m)

Anterior-posterior axis (m)

Anterior-posterior axis (m)

Anterior-posterior axis (m)

Anterior-posterior axis (m)

Anterior-posterior axis (m)

Anterior-posterior axis (m)

Anterior-posterior axis (m)

Anterior-posterior axis (m)

Anterior-posterior axis (m)

Anterior-posterior axis (m)

Anterior-posterior axis (m)

Anterior-posterior axis (m)

Anterior-posterior axis (m)

Anterior-posterior axis (m)

Anterior-posterior axis (m)

Anterior-posterior axis (m)

Anterior-posterior axis (m)

Anterior-posterior axis (m)

Anterior-posterior axis (m)

Anterior-posterior axis (m)

Anterior-posterior axis (m)

Anterior-posterior axis (m)

Anterior-posterior axis (m)

Anterior-posterior axis (m)

Anterior-posterior axis (m)

Anterior-posterior axis (m)

Anterior-posterior axis (m)

Anterior-posterior axis (m)

Anterior-posterior axis (m)

Anterior-posterior axis (m)

Anterior-posterior axis (m)

Anterior-posterior axis (m)

Anterior-posterior axis (m)

Anterior-posterior axis (m)

Anterior-posterior axis (m)

Anterior-posterior axis (m)

Anterior-posterior axis (m)

Anterior-posterior axis (m)

Anterior-posterior axis (m)

Anterior-posterior axis (m)

Anterior-posterior axis (m)

Anterior-posterior axis (m)

Anterior-posterior axis (m)

Anterior-posterior axis (m)

Anterior-posterior axis (m)

Anterior-posterior axis (m)

Anterior-posterior axis (m)

Anterior-posterior axis (m)

Anterior-posterior axis (m)

Anterior-posterior axis (m)

Anterior-posterior axis (m)

Anterior-posterior axis (m)

Anterior-posterior axis (m)

Anterior-posterior axis (m)

Anterior-posterior axis (m)

Anterior-posterior axis (m)

Anterior-posterior axis (m)

Anterior-posterior axis (m)

Anterior-posterior axis (m)

Anterior-posterior axis (m)

Anterior-posterior axis (m)

Anterior-posterior axis (m)

Anterior-posterior axis (m)

Anterior-posterior axis (m)

Anterior-posterior axis (m)

Anterior-posterior axis (m)

Anterior-posterior axis (m)

Anterior-posterior axis (m)

Anterior-posterior axis (m)

Anterior-posterior axis (m)

Anterior-posterior axis (m)

Anterior-posterior axis (m)

Anterior-posterior axis (m)

Anterior-posterior axis (m)

Anterior-posterior axis (m)

Anterior-posterior axis (m)

Anterior-posterior axis (m)

Anterior-posterior axis (m)

Anterior-posterior axis (m)

Anterior-posterior axis (m)

Anterior-posterior axis (m)

Anterior-posterior axis (m)

Anterior-posterior axis (m)

Anterior-posterior axis (m)

Anterior-posterior axis (m)

Anterior-posterior axis (m)

Anterior-posterior axis (m)

Anterior-posterior axis (m)

Anterior-posterior axis (m)

Anterior-posterior axis (m)

Anterior-posterior axis (m)

Anterior-posterior axis (m)

Anterior-posterior axis (m)

Anterior-posterior axis (m)

Anterior-posterior axis (m)

Anterior-posterior axis (m)

Anterior-posterior axis (m)

Anterior-posterior axis (m)

Anterior-posterior axis (m)

Anterior-posterior axis (m)

Anterior-posterior axis (m)

Anterior-posterior axis (m)

Anterior-posterior axis (m)

Anterior-posterior axis (m)

Anterior-posterior axis (m)

Anterior-posterior axis (m)

Anterior-posterior axis (m)

Anterior-posterior axis (m)

Anterior-posterior axis (m)

Anterior-posterior axis (m)

Anterior-posterior axis (m)

Anterior-posterior axis (m)

Anterior-posterior axis (m)

Anterior-posterior axis (m)

Anterior-posterior axis (m)

Anterior-posterior axis (m)

Anterior-posterior axis (m)

Anterior-posterior axis (m)

Anterior-posterior axis (m)

Anterior-posterior axis (m)

Anterior-posterior axis (m)

Anterior-posterior axis (m)

Anterior-posterior axis (m)

Anterior-posterior axis (m)

Anterior-posterior axis (m)

Anterior-posterior axis (m)

Anterior-posterior axis (m)

Anterior-posterior axis (m)

Anterior-posterior axis (m)

Anterior-posterior axis (m)

Anterior-posterior axis (m)

Anterior-posterior axis (m)

Anterior-posterior axis (m)

Anterior-posterior axis (m)

Anterior-posterior axis (m)

Anterior-posterior axis (m)

Anterior-posterior axis (m)

Anterior-posterior axis (m)

Anterior-posterior axis (m)

Anterior-posterior axis (m)

Anterior-posterior axis (m)

Anterior-posterior axis (m)

Anterior-posterior axis (m)

Anterior-posterior axis (m)

Anterior-posterior axis (m)

Anterior-posterior axis (m)

Anterior-posterior axis (m)

Anterior-posterior axis (m)

Anterior-posterior axis (m)

Anterior-posterior axis (m)

Anterior-posterior axis (m)

Anterior-posterior axis (m)

Anterior-posterior axis (m)

Anterior-posterior axis (m)

Anterior-posterior axis (m)

Anterior-posterior axis (m)

Anterior-posterior axis (m)

Anterior-posterior axis (m)

Anterior-posterior axis (m)

Anterior-posterior axis (m)

Anterior-posterior axis (m)

Anterior-posterior axis (m)

Anterior-posterior axis (m)

Anterior-posterior axis (m)

Anterior-posterior axis (m)

Anterior-posterior axis (m)

Anterior-posterior axis (m)

Anterior-posterior axis (m)

Anterior-posterior axis (m)

Anterior-posterior axis (m)

Anterior-posterior axis (m)

Anterior-posterior axis (m)

Anterior-posterior axis (m)

Anterior-posterior axis (m)

Anterior-posterior axis (m)

Anterior-posterior axis (m)

Anterior-posterior axis (m)

Anterior-posterior axis (m)

Anterior-posterior axis (m)

Anterior-posterior axis (m)

Anterior-posterior axis (m)

Anterior-posterior axis (m)

Anterior-posterior axis (m)

Anterior-posterior axis (m)

Anterior-posterior axis (m)

Anterior-posterior axis (m)

Anterior-posterior axis (m)

Anterior-posterior axis (m)

Anterior-posterior axis (m)

Anterior-posterior axis (m)

Anterior-posterior axis (m)

Anterior-posterior axis (m)

Anterior-posterior axis (m)

Anterior-posterior axis (m)

Anterior-posterior axis (m)

Anterior-posterior axis (m)

Anterior-posterior axis (m)

Anterior-posterior axis (m)

Anterior-posterior axis (m)

Anterior-posterior axis (m)

Anterior-posterior axis (m)

Anterior-posterior axis (m)

Anterior-posterior axis (m)

Anterior-posterior axis (m)

Anterior-posterior axis (m)

Anterior-posterior axis (m)

Anterior-posterior axis (m)

Anterior-posterior axis (m)

Anterior-posterior axis (m)

Anterior-posterior axis (m)

Anterior-posterior axis (m)

Anterior-posterior axis (m)

Anterior-posterior axis (m)

Anterior-posterior axis (m)

Anterior-posterior axis (m)

Anterior-posterior axis (m)

Anterior-posterior axis (m)

Anterior-posterior axis (m)

Anterior-posterior axis (m)

Anterior-posterior axis (m)

Anterior-posterior axis (m)

Anterior-posterior axis (m)

Anterior-posterior axis (m)

Anterior-posterior axis (m)

Anterior-posterior axis (m)

Anterior-posterior axis (m)

Anterior-posterior axis (m)

Anterior-posterior axis (m)

Anterior-posterior axis (m)

Anterior-posterior axis (m)

Anterior-posterior axis (m)

Anterior-posterior axis (m)

Anterior-posterior axis (m)

Anterior-posterior axis (m)

Anterior-posterior axis (m)

Anterior-posterior axis (m)

Anterior-posterior axis (m)

Anterior-posterior axis (m)

Anterior-posterior axis (m)

Anterior-posterior axis (m)

Anterior-posterior axis (m)

Anterior-posterior axis (m)

Anterior-posterior axis (m)

Anterior-posterior axis (m)

Anterior-posterior axis (m)

Anterior-posterior axis (m)

Anterior-posterior axis (m)

Anterior-posterior axis (m)

Anterior-posterior axis (m)

Anterior-posterior axis (m)

Anterior-posterior axis (m)

Anterior-posterior axis (m)

Anterior-posterior axis (m)

Anterior-posterior axis (m)

Anterior-posterior axis (m)

Anterior-posterior axis (m)

Anterior-posterior axis (m)

Anterior-posterior axis (m)

Anterior-posterior axis (m)

Anterior-posterior axis (m)

Anterior-posterior axis (m)

Anterior-posterior axis (m)

Anterior-posterior axis (m)

Anterior-posterior axis (m)

Anterior-posterior axis (m)

Anterior-posterior axis (m)

Anterior-posterior axis (m)

Anterior-posterior axis (m)

Anterior-posterior axis (m)

Anterior-posterior axis (m)

Anterior-posterior axis (m)

Anterior-posterior axis (m)

Anterior-posterior axis (m)

Anterior-posterior axis (m)

Anterior-posterior axis (m)

Anterior-posterior axis (m)

Anterior-posterior axis (m)

Anterior-posterior axis (m)

Anterior-posterior axis (m)

Anterior-posterior axis (m)

Anterior-posterior axis (m)

Anterior-posterior axis (m)

Anterior-posterior axis (m)

Anterior-posterior axis (m)

Anterior-posterior axis (m)

Anterior-posterior axis (m)

Anterior-posterior axis (m)

Anterior-posterior axis (m)

Anterior-posterior axis (m)

Anterior-posterior axis (m)

Anterior-posterior axis (m)

Anterior-posterior axis (m)

Anterior-posterior axis (m)

Anterior-posterior axis (m)

Anterior-posterior axis (m)

Anterior-posterior axis (m)

Anterior-posterior axis (m)

Anterior-posterior axis (m)

Anterior-posterior axis (m)

Anterior-posterior axis (m)

Anterior-posterior axis (m)

Anterior-posterior axis (m)

Anterior-posterior axis (m)

Anterior-posterior axis (m)

Anterior-posterior axis (m)

Anterior-posterior axis (m)

Anterior-posterior axis (m)

Anterior-posterior axis (m)

Anterior-posterior axis (m)

Anterior-posterior axis (m)

Anterior-posterior axis (m)

Anterior-posterior axis (m)

Anterior-posterior axis (m)

Anterior-posterior axis (m)

Anterior-posterior axis (m)

Anterior-posterior axis (m)

Anterior-posterior axis (m)

Anterior-posterior axis (m)

Anterior-posterior axis (m)

Anterior-posterior axis (m)

Anterior-posterior axis (m)

Anterior-posterior axis (m)

Anterior-posterior axis (m)

Anterior-posterior axis (m)

Anterior-posterior axis (m)

Anterior-posterior axis (m)

Anterior-posterior axis (m)

Anterior-posterior axis (m)

Anterior-posterior axis (m)

Anterior-posterior axis (m)

Anterior-posterior axis (m)

Anterior-posterior axis (m)

Anterior-posterior axis (m)

Anterior-posterior axis (m)

Anterior-posterior axis (m)

Anterior-posterior axis (m)

Anterior-posterior axis (m)

Anterior-posterior axis (m)

Anterior-posterior axis (m)

Anterior-posterior axis (m)

Anterior-posterior axis (m)

Anterior-posterior axis (m)

Anterior-posterior axis (m)

Anterior-posterior axis (m)

Anterior-posterior axis (m)

Anterior-posterior axis (m)

Anterior-posterior axis (m)

Anterior-posterior axis (m)

Anterior-posterior axis (m)

Anterior-posterior axis (m)

Anterior-posterior axis (m)

Anterior-posterior axis (m)

Anterior-posterior axis (m)

Anterior-posterior axis (m)

Anterior-posterior axis (m)

Anterior-posterior axis (m)

Anterior-posterior axis (m)

Anterior-posterior axis (m)

Anterior-posterior axis (m)

Anterior-posterior axis (m)

Anterior-posterior axis (m)

Anterior-posterior axis (m)

Anterior-posterior axis (m)

Anterior-posterior axis (m)

Anterior-posterior axis (m)

Anterior-posterior axis (m)

Anterior-posterior axis (m)

Anterior-posterior axis (m)

Anterior-posterior axis (m)

Anterior-posterior axis (m)

Anterior-posterior axis (m)

Anterior-posterior axis (m)

Anterior-posterior axis (m)

Anterior-posterior axis (m)

Anterior-posterior axis (m)

Anterior-posterior axis (m)

Anterior-posterior axis (m)

Anterior-posterior axis (m)

Anterior-posterior axis (m)

Anterior-posterior axis (m)

Anterior-posterior axis (m)

Anterior-posterior axis (m)

Anterior-posterior axis (m)

Anterior-posterior axis (m)

Anterior-posterior axis (m)

Anterior-posterior axis (m)

Anterior-posterior axis (m)

Anterior-posterior axis (m)

Anterior-posterior axis (m)

Anterior-posterior axis (m)

Anterior-posterior axis (m)

Anterior-posterior axis (m)

Anterior-posterior axis (m)

Anterior-posterior axis (m)

Anterior-posterior axis (m)

Anterior-posterior axis (m)

Anterior-posterior axis (m)

Anterior-posterior axis (m)

Anterior-posterior axis (m)

Anterior-posterior axis (m)

Anterior-posterior axis (m)

Anterior-posterior axis (m)

Anterior-posterior axis (m)

Anterior-posterior axis (m)

Anterior-posterior axis (m)

Anterior-posterior axis (m)

Anterior-posterior axis (m)

Anterior-posterior axis (m)

Anterior-posterior axis (m)

Anterior-posterior axis (m)

Anterior-posterior axis (m)

Anterior-posterior axis (m)

Anterior-posterior axis (m)

Anterior-posterior axis (m)

Anterior-posterior axis (m)

Anterior-posterior axis (m)

Anterior-posterior axis (m)

Anterior-posterior axis (m)

Anterior-posterior axis (m)

Anterior-posterior axis (m)

Anterior-posterior axis (m)

Anterior-posterior axis (m)

Anterior-posterior axis (m)

Anterior-posterior axis (m)

Anterior-posterior axis (m)

Anterior-posterior axis (m)

Anterior-posterior axis (m)

Anterior-posterior axis (m)

Anterior-posterior axis (m)

Anterior-posterior axis (m)

Anterior-posterior axis (m)

Anterior-posterior axis (m)

Anterior-posterior axis (m)

Anterior-posterior axis (m)

Anterior-posterior axis (m)

Anterior-posterior axis (m)

Anterior-posterior axis (m)

Anterior-posterior axis (m)

Anterior-posterior axis (m)

Anterior-posterior axis (m)

Anterior-posterior axis (m)

Anterior-posterior axis (m)

Anterior-posterior axis (m)

Anterior-posterior axis (m)

Anterior-posterior axis (m)

Anterior-posterior axis (m)

Anterior-posterior axis (m)

Anterior-posterior axis (m)

Anterior-posterior axis (m)

Anterior-posterior axis (m)

Anterior-posterior axis (m)

Anterior-posterior axis (m)

Anterior-posterior axis (m)

Anterior-posterior axis (m)

Anterior-posterior axis (m)

Anterior-posterior axis (m)

Anterior-posterior axis (m)

Anterior-posterior axis (m)

Anterior-posterior axis (m)

Anterior-posterior axis (m)

Anterior-posterior axis (m)

Anterior-posterior axis (m)

Anterior-posterior axis (m)

Anterior-posterior axis (m)

Anterior-posterior axis (m)

Anterior-posterior axis (m)

Anterior-posterior axis (m)

Anterior-posterior axis (m)

Anterior-posterior axis (m)

Anterior-posterior axis (m)

Anterior-posterior axis (m)

Anterior-posterior axis (m)

Anterior-posterior axis (m)

Anterior-posterior axis (m)

Anterior-posterior axis (m)

Anterior-posterior axis (m)

Anterior-posterior axis (m)

Anterior-posterior axis (m)

Anterior-posterior axis (m)

Anterior-posterior axis (m)

Anterior-posterior axis (m)

Anterior-posterior axis (m)

Anterior-posterior axis (m)

Anterior-posterior axis (m)

Anterior-posterior axis (m)

Anterior-posterior axis (m)

Anterior-posterior axis (m)

Anterior-posterior axis (m)

Anterior-posterior axis (m)

Anterior-posterior axis (m)

Anterior-posterior axis (m)

Anterior-posterior axis (m)

Anterior-posterior axis (m)

Anterior-posterior axis (m)

Anterior-posterior axis (m)

Anterior-posterior axis (m)

Anterior-posterior axis (m)

Anterior-posterior axis (m)

Anterior-posterior axis (m)

Anterior-posterior axis (m)

Anterior-posterior axis (m)

Anterior-posterior axis (m)

Anterior-posterior axis (m)

Anterior-posterior axis (m)

Anterior-posterior axis (m)

Anterior-posterior axis (m)

Anterior-posterior axis (m)

Anterior-posterior axis (m)

Anterior-posterior axis (m)

Anterior-posterior axis (m)

Anterior-posterior axis (m)

Anterior-posterior axis (m)

Anterior-posterior axis (m)

Anterior-posterior axis (m)

Anterior-posterior axis (m)

Anterior-posterior axis (m)

Anterior-posterior axis (m)

Anterior-posterior axis (m)

Anterior-posterior axis (m)

Anterior-posterior axis (m)

Anterior-posterior axis (m)

Anterior-posterior axis (m)

Anterior-posterior axis (m)

Anterior-posterior axis (m)

Anterior-posterior axis (m)

Anterior-posterior axis (m)

Anterior-posterior axis (m)

Anterior-posterior axis (m)

Anterior-posterior axis (m)

Anterior-posterior axis (m)

Anterior-posterior axis (m)

Anterior-posterior axis (m)

Anterior-posterior axis (m)

Anterior-posterior axis (m)

Anterior-posterior axis (m)

Anterior-posterior axis (m)

Anterior-posterior axis (m)

Anterior-posterior axis (m)

Anterior-posterior axis (m)

Anterior-posterior axis (m)

Anterior-posterior axis (m)

Anterior-posterior axis (m)

Anterior-posterior axis (m)

Anterior-posterior axis (m)

Anterior-posterior axis (m)

Anterior-posterior axis (m)

Anterior-posterior axis (m)

Anterior-posterior axis (m)

Anterior-posterior axis (m)

Anterior-posterior axis (m)

Anterior-posterior axis (m)

Anterior-posterior axis (m)

Anterior-posterior axis (m)

Anterior-posterior axis (m)

Anterior-posterior axis (m)

Anterior-posterior axis (m)

Anterior-posterior axis (m)

Anterior-posterior axis (m)

Anterior-posterior axis (m)

Anterior-posterior axis (m)

Anterior-posterior axis (m)

Anterior-posterior axis (m)

Anterior-posterior axis (m)

Anterior-posterior axis (m)

Anterior-posterior axis (m)

Anterior-posterior axis (m)

Anterior-posterior axis (m)

Anterior-posterior axis (m)

Anterior-posterior axis (m)

Anterior-posterior axis (m)

Anterior-posterior axis (m)

Anterior-posterior axis (m)

Anterior-posterior axis (m)

Anterior-posterior axis (m)

Anterior-posterior axis (m)

Anterior-posterior axis (m)

Anterior-posterior axis (m)

Anterior-posterior axis (m)

Anterior-posterior axis (m)

Anterior-posterior axis (m)

Anterior-posterior axis (m)

Anterior-posterior axis (m)

Anterior-posterior axis (m)

Anterior-posterior axis (m)

Anterior-posterior axis (m)

Anterior-posterior axis (m)

Anterior-posterior axis (m)

Anterior-posterior axis (m)

Anterior-posterior axis (m)

Anterior-posterior axis (m)

Anterior-posterior axis (m)

Anterior-posterior axis (m)

Anterior-posterior axis (m)

Anterior-posterior axis (m)

Anterior-posterior axis (m)

Anterior-posterior axis (m)

Anterior-posterior axis (m)

Anterior-posterior axis (m)

Anterior-posterior axis (m)

Anterior-posterior axis (m)

Anterior-posterior axis (m)

Anterior-posterior axis (m)

Anterior-posterior axis (m)

Anterior-posterior axis (m)

Anterior-posterior axis (m)

Anterior-posterior axis (m)

Anterior-posterior axis (m)

Anterior-posterior axis (m)

Anterior-posterior axis (m)

Anterior-posterior axis (m)

Anterior-posterior axis (m)

Anterior-posterior axis (m)

Anterior-posterior axis (m)

Anterior-posterior axis (m)

Anterior-posterior axis (m)

Anterior-posterior axis (m)

Anterior-posterior axis (m)

Anterior-posterior axis (m)

Anterior-posterior axis (m)

Anterior-posterior axis (m)

Anterior-posterior axis (m)

Anterior-posterior axis (m)

Anterior-posterior axis (m)

Anterior-posterior axis (m)

Anterior-posterior axis (m)

Anterior-posterior axis (m)

Anterior-posterior axis (m)

Anterior-posterior axis (m)

Anterior-posterior axis (m)

Anterior-posterior axis (m)

Anterior-posterior axis (m)

Anterior-posterior axis (m)

Anterior-posterior axis (m)

Anterior-posterior axis (m)

Anterior-posterior axis (m)

Anterior-posterior axis (m)

Anterior-posterior axis (m)

Anterior-posterior axis (m)

Anterior-posterior axis (m)

Anterior-posterior axis (m)

Anterior-posterior axis (m)

Anterior-posterior axis (m)

Anterior-posterior axis (m)

Anterior-posterior axis (m)

Anterior-posterior axis (m)

Anterior-posterior axis (m)

Anterior-posterior axis (m)

Anterior-posterior axis (m)

Anterior-posterior axis (m)

Anterior-posterior axis (m)

Anterior-posterior axis (m)

Anterior-posterior axis (m)

Anterior-posterior axis (m)

Anterior-posterior axis (m)

Anterior-posterior axis (m)

Anterior-posterior axis (m)

Anterior-posterior axis (m)

Anterior-posterior axis (m)

Calculation method for the onset of reorientation strategy during the shifting condition. The displacement trajectory of the individual is shown in purple, with target shifting condition to the right. The result (time of onset of re-orientation) is illustrated by the red star. This point is found by fitting the displacement trajectory lines pre- and post-target shift point (that occurs at 1.5 meters of forward displacement, illustrated by bleu lines) and determining the intercept point of these two lines (i.e. red star).
